# Gut Mycobiota‐Associated Tryptophan Catabolites Protect Against Metabolic Dysfunction‐Associated Steatotic Liver Disease

**DOI:** 10.1002/advs.202514830

**Published:** 2026-04-29

**Authors:** Shuping Qiao, Shuangya Fan, Juan Xu, Zhen Xu, Chen Peng, Junxing Qu, Ziqian Bing, Shizhen Zhou, Sunan Shen, Guifang Xu, Yue Zhao, Tingting Wang

**Affiliations:** ^1^ The State Key Laboratory of Pharmaceutical Biotechnology Chemistry and Biomedicine Innovation Center (ChemBIC) Division of Immunology Medical School Nanjing University Nanjing Jiangsu China; ^2^ Jiangsu Key Laboratory of Molecular Medicine Division of Immunology Medical School Nanjing University Nanjing Jiangsu China; ^3^ Hospital of Integrated Traditional Chinese and Western Medicine Nanjing University of Chinese Medicine Nanjing Jiangsu China; ^4^ The Comprehensive Cancer Centre of Nanjing Drum Tower Hospital Affiliated Hospital of Nanjing University Medical School Nanjing Jiangsu China; ^5^ Institutes of Health Central Plains Xinxiang Medical University Xinxiang Henan China; ^6^ Xinxiang Key Laboratory for Tumor Drug Screening and Targeted Therapy Xinxiang Henan China; ^7^ Department of General Surgery Nanjing Drum Tower Hospital Affiliated Hospital of Medical School Nanjing University Nanjing Jiangsu China; ^8^ Department of Gastroenterology The First Affiliated Hospital of Anhui Medical University Hefei Anhui China

**Keywords:** fatty acid oxidation, fungi, host–microbe interaction, metabolic associated steatotic liver disease, tryptophan metabolism

## Abstract

Accumulating evidence suggests that the intestinal microbiota participates in the progression of metabolic dysfunction‐associated steatotic liver disease (MASLD) through microbiota‐host interaction. However, the beneficial role of commensal mycobiota in MASLD progression remains poorly understood. By comparing the gut microbiome differences, we demonstrated that the deficiency of Caspase Recruitment Domain‐containing protein 9 (CARD9), an adaptor protein for a microbiota recognition receptor, exacerbated high‐fat diet (HFD)‐induced MASLD in a gut fungi‐dependent manner. CARD9 deficiency reduced the abundance of *Saccharomyces cerevisiae* (*S. cerevisiae*), which was a probiotic alleviating MASLD progression. *S. cerevisiae* promoted a significantly greater abundance of 5‐hydroxyindoleacetic acid (5‐HIAA) in the intestine through Toll‐like receptor 1 (TLR1), which reduced body weight in mice and alleviated MASLD phenotypes via the “gut‐liver” axis. Particularly, 5‐HIAA directly binds to aryl‐hydrocarbon receptor (AhR) and stimulates its nuclear translocation, subsequently inducing fatty acid oxidation via carnitine palmitoyltransferase 1A (CPT1A) and acyl‐CoA oxidase 1 (ACOX1) transactivation. MASLD patients exhibited decreased levels of *S. cerevisiae* and 5‐HIAA, and *S. cerevisiae* effectively reduced hepatic steatosis and improved glucose homeostasis in patients with MASLD. In summary, our findings identified a novel pathway of fungi‐*S. cerevisiae* stimulating intestinal 5‐HIAA production and indicated that *S. cerevisiae* and 5‐HIAA might alleviate MASLD progression, highlighting that the mycobiota‐dependent gut‐liver axis was a promising target for the prevention of MASLD.

## Graphical abstract

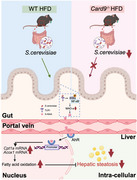

## 1 Introduction

Metabolic dysfunction‐associated steatotic liver disease (MASLD) is considered a metabolic syndrome, which is highly prevalent among individuals with obesity and type 2 diabetes mellitus [1]. It is characterized by steatosis, indicated by triglyceride (TG) accumulation in over 5% of hepatocytes, occurring in the absence of alcohol intake [2]. Genetic factors, metabolic factors, and dysbiosis of intestinal flora have important roles in the development of MASLD [3, 4, 5]. Therefore, elucidating the pathogenesis of MASLD can provide a theoretical basis for clinical treatment.

More evidence indicates that dysbiosis of the intestinal flora is generally involved in the pathogenesis of MASLD [6, 7, 8], accompanied by changes in microbial‐derived metabolites [9]. Previous studies have shown that probiotics and commensal gut bacteria are capable of modulating the composition of the intestinal microbiota [10, 11], reducing hepatic triglyceride levels [11], enhancing mucosal barrier function [12], and activating the host immune system [13]. Several bacteria‐derived metabolites, such as short‐chain fatty acids (SCFAs), bile acids, and indole, regulate insulin sensitivity and inflammation of the liver [6, 14, 15, 16]. Besides bacteria, mycobiota is also an important member of gut microbiota. Recent work has shown that the fungal composition in the feces of MASLD patients differs from that of healthy individuals, with an increase in *Candida albicans (C. albicans)* [10]. Besides, *C. albicans*‐reactive T helper 17 (Th17) cells migrate from the intestine to the liver to aggravate Alcoholic fatty liver disease [17]. Moreover, recent work has reported intestinal filamentous fungi‐*Fusarium* spp. reversed Metabolic dysfunction‐associated steatohepatitis (MASH) progression through a secondary metabolite [18]. Nonetheless, the contribution of the commensal fungal microbiota to the progression of MASLD remains unclear.

As a key adaptor protein, CARD9 is mainly present in myeloid cells [19, 20]. After activation of C‐type lectin receptor (CLR), CARD9 can generate CARD9‐BCL10‐MALT1 (CBM) complex with B cell leukemia‐lymphoma 10 (Bcl10) and mucosa‐associated lymphoid tissue (Malt1). This complex further activates inflammatory response pathways such as nuclear factor kappa B (NF‐κB) and mitogen‐activated protein kinases (MAPK) to promote the production and release of inflammatory cytokines in macrophages [21]. CARD9 can activate the immune cascade and assist Th17 cells to produce IL‐17, which is critically involved in inflammatory bowel disease and fungal infectious diseases [22]. Our earlier studies have shown an increased *Candida. tropicalis* burden in the gut of *Card9*‐deficient (*Card9* ^−^) mice, resulting in intestinal barrier dysfunction [23]. Deletion of hematopoietic *Card9* increased atherosclerotic lesion formation and thus atherosclerosis development [24], indicating functional interactions of CARD9 with commensal fungi are crucial for metabolic homeostasis. Although *Card9* ^−^ mice have been shown to be more vulnerable to fungal infections, such as *C. albicans* [25], the impact of CARD9‐mediated gut mycobiota dysbiosis on MASLD development is poorly understood.

Increasing studies demonstrate that tryptophan (Trp), along with its bioactive metabolites generated by gut microbes, is associated with type 2 diabetes [26]. There are three principal pathways governing intestinal Trp metabolism [27]: the indole/aryl hydrocarbon receptor (AhR) pathway, the Kynurenine (Kyn) pathway, and the serotonin pathway. Although it has been reported that gut bacteria can directly metabolize tryptophan into 5‐hydroxyindoleacetic acid (5‐HIAA) [28], the evidence for how probiotic fungi modulate host tryptophan metabolism to prevent MASLD is limited.

Here, we assessed the effect of mycobiota dysbiosis on MASLD progression and found that *Card9* ^−^ mice with gut mycobiota dysbiosis displayed increased hepatic steatosis under a high‐fat diet (HFD). Microbiome profiling revealed that a probiotic fungi‐*Saccharomyces cerevisiae* (*S. cerevisiae*) was lower in feces of HFD‐*Card9* ^−^ mice and MASLD patients. Supplementation with *S. cerevisiae* alleviated liver steatosis by enhancing fatty acid oxidation (FAO). Furthermore, we confirmed that *S. cerevisiae* promoted a significantly greater abundance of 5‐HIAA in the intestine, which then alleviated MASLD phenotypes via the “gut‐liver” axis. Particularly, 5‐HIAA directly bound to AhR and subsequently stimulated carnitine palmitoyltransferase 1A (CPT1A) and acyl‐CoA oxidase 1 (ACOX1)‐dependent fatty acid oxidation. Importantly, *S. cerevisiae* effectively ameliorated hepatic steatosis and improved glucose homeostasis in patients with MASLD. These results elucidated the mechanism of a new gut mycobiota‐dependent tryptophan metabolic signaling axis between the gut and the liver, which provided a new therapeutic strategy for MASLD.

## 2 Results

### 2.1 *Card9* ^−^ Mice Have Severe MASLD Upon HFD Treatment Than WT Mice

**FIGURE 1:**
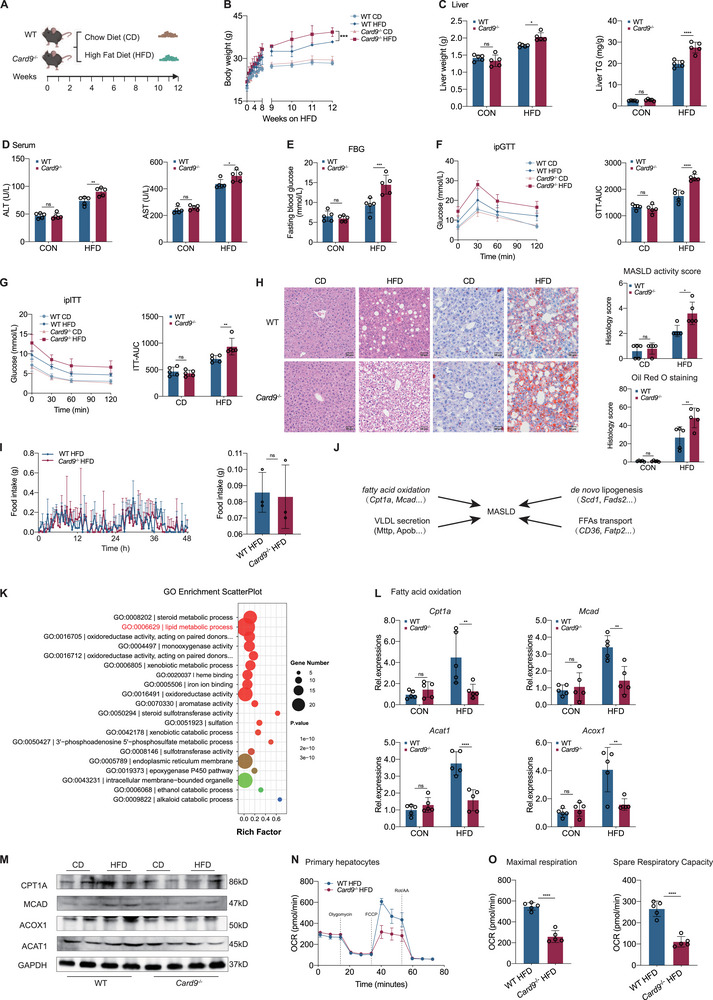
*Card9* ^−^ mice exhibit severe MASLD upon HFD treatment. (A) Experimental schematic of WT and *Card9* ^−^ mice fed with Chow Diet (CD)/high‐fat diet (HFD) for 12 weeks. (B) Body weight changes during the 12‐week experimental period. (C) Liver weight of mice in different groups (left) and content of Triglyceride (TG) in the liver of mice in different groups (right). (D) Levels of ALT and AST in serum. (E) The glucose concentration at fasting levels. (F) Levels of serum glucose for intraperitoneal glucose tolerance tests (ipGTT) (left) and area under the curve (AUC) (right). (G) Levels of serum glucose for intraperitoneal insulin tolerance tests (ipITT) (left) and area under the curve (AUC) (right). (H) Representative liver histology by H&E staining (left) and Oil Red O staining (right). Scale bars = 50 µm. MASLD activity score quantification (up) and Oil Red O positive area quantification (down). (I) Food intake within 48h (left) and average food intake within 48h (right). (J) Experimental schematic of the lipid metabolism process associated with MASLD. (K) Differentially expressed genes (DEGs) between HFD‐fed WT mice liver and HFD‐fed *Card9* ^−^ mice liver were subjected to GO enrichment analysis. (L) Q‐PCR analysis of relative mRNA expression levels of genes involved in fatty acid oxidation (*Cpt1a*, *Mcad*, *Acat1*, *Acox1*) in mice livers. (M) Western blot analysis of relative protein involved in fatty acid oxidation (CPT1A, MCAD, ACOX1, ACAT1) in mice livers. GAPDH was used as the internal standard. (N) Oxygen consumption rate of HFD‐fed WT mice and HFD‐fed *Card9* ^−^ mice livers measured by Seahorse. (O) Individual parameters for maximum respiration and spare respiratory capacity were measured by Seahorse. Each dot represents data from an individual biological replicate. Biological replicate numbers in each group are as follows: (figure 1B–H, L–O) *n* = 5, (figure 1I) *n* = 3, (figure 1K) *n* = 6. Data were shown as mean ± SEM. ^*^ *p* < 0.05, ^**^ *p* < 0.01, ^***^ *p* < 0.001 as determined by two‐way ANOVA followed by Tukey's multiple comparison (B–H, L) or two‐tailed, unpaired Student's t‐test (I, O).

To determine the potential effect of CARD9 in MASLD, we fed WT and *Card9* ^−^ mice with high‐fat diet (HFD) for 12 weeks (Figure 1A), which recapitulates obesity‐associated MASLD. We found that the body weight was increased in HFD‐fed *Card9* ^−^ mice compared with HFD‐fed WT mice (Figure 1B). The liver weight, liver triglyceride (TG) content (Figure 1C), alanine aminotransferase (ALT), aspartate aminotransferase (AST) (Figure 1D), and the glucose concentration at fasting levels (Figure 1E) were all significantly raised in HFD‐fed *Card9* ^−^ mice relative to HFD‐fed WT mice. Furthermore, we found HFD‐fed *Card9* ^−^ mice showed higher blood glucose levels and impaired insulin sensitivity compared with HFD‐fed WT mice during the intraperitoneal glucose tolerance test (ipGTT) and the intraperitoneal insulin tolerance test (ipITT) (Figure 1F,G). Moreover, the levels of total cholesterol (TC), triglyceride (TG), and non‐esterified fatty acids (NEFA) in serum (Figure S1A) were more aggravated in HFD‐fed *Card9* ^−^ mice relative to HFD‐fed WT mice. As assessed by hematoxylin and eosin (H&E) and Oil red O staining, *Card9* ^−^ mice exhibited more severe MASLD symptoms under HFD treatment, as indicated by increased MASLD activity score and hepatic steatosis (Figure 1H). Moreover, HFD‐fed *Card9* ^−^ mice showed profound steatosis with respect to HFD‐fed WT mice, as evidenced by liver size and hepatic lipid accumulation (Figure S1B,C).

To explore why HFD‐fed *Card9* ^−^ mice have more severe MASLD than HFD‐fed WT mice, we measured food intake in HFD mice within 48 h (Figure 1I). No obvious difference in food intake was observed during this period. Hepatic TG content is primarily influenced by the absorption of free fatty acids (FFAs) from white adipose tissue [29] as well as by *de novo* lipid synthesis [30, 31], which is balanced by fatty acid breakdown through β‐oxidation [32, 33], and secretion of very low‐density lipoprotein (VLDL) [34] (Figure 1J). MASLD develops when the processes of fatty acid breakdown and lipid secretion are insufficient to balance the elevated lipid absorption and production. We conducted RNA‐seq analysis for livers derived from HFD‐fed WT mice and HFD‐fed *Card9* ^−^ mice. We observed significant alterations in lipid metabolism processes (Figure 1K). Then we found the fatty acid oxidation‐related genes and proteins, such as *carnitine palmitoyltransferase 1a* (*Cpt1a*), *medium‐chain acyl‐CoA dehydrogenase* (*Mcad*), *acetyl‐CoA acetyltransferase 1* (Acat1), and *acyl‐CoA oxidase 1* (*Acox1*), were more significantly downregulated in livers of HFD‐fed *Card9* ^−^ mice than in HFD‐fed WT mice (Figure 1L,M). Furthermore, hepatocytes from HFD‐fed *Card9* ^−^ mice exhibited decreased mitochondrial maximal respiration and spare respiratory capacity compared with HFD‐fed WT mice (Figure 1N,O). However, the expressions of genes associated with *de novo* lipogenesis, VLDL secretion, and FFAs transport showed no meaningful difference between HFD‐fed *Card9* ^−^ mice and HFD‐fed WT mice (Figure S1D–F). Thus, *Card9*‐deficient mice fed an HFD exhibited more severe hepatic steatosis together with reduced fatty acid oxidation.

### 2.2 Accelerated MASLD in HFD‐fed *Card9* ^−^ Mice Relies on Intestinal Fungus

**FIGURE 2:**
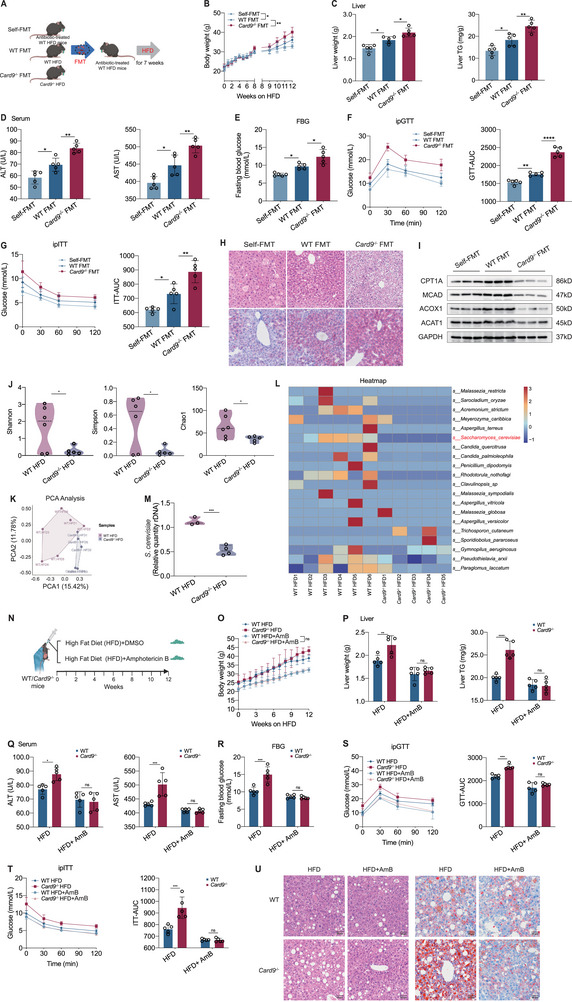
Accelerated MASLD in HFD‐fed *Card9* ^−^ mice relies on intestinal fungus. (A) Experimental schematic of the Fecal Microbiota Transplantation model. (B) Body weight changes during the 12‐week experimental period. (C) Liver weight of mice in different groups (left) and content of Triglyceride (TG) in the liver of mice in different groups (right). (D) Levels of ALT and AST in serum. (E) The glucose concentration at fasting levels. (F) Levels of serum glucose for intraperitoneal glucose tolerance tests (ipGTT) (left) and area under the curve (AUC) (right). (G) Levels of serum glucose for intraperitoneal insulin tolerance tests (ipITT) (left) and area under the curve (AUC) (right). (H) Representative liver histology by H&E staining (up) and Oil Red O staining (down). Scale bars = 50 µm. (I) Western blot analysis of relative protein involved in fatty acid oxidation (CPT1A, MCAD, ACOX1, ACAT1) in mice livers. GAPDH was used as the internal standard. (J) α‐diversity analysis (Shannon, Simpson, and Chao1) based on fungal ITS2 gene sequence abundance in the feces. (K) Weighted UniFrac PCA analysis. (L) The relative abundances of different fungal species based on the fungal ITS2 gene sequence. (M) The burden of *S. cerevisiae* in the feces of HFD mice. (N) Experimental schematic of WT and *Card9* *−* mice fed with high‐fat diet (HFD) for 12 weeks with/without Amphotericin B treatment. (O) Body weight changes during the 12‐week experimental period. (P) Liver weight of mice in different groups (left) and content of Triglyceride (TG) in the liver in different groups (right). (Q) Levels of ALT and AST in serum. (R) The glucose concentration at fasting levels. (S) Levels of serum glucose for intraperitoneal glucose tolerance tests (ipGTT) (left) and area under the curve (AUC) (right). (T) Levels of serum glucose for intraperitoneal insulin tolerance tests (ipITT) (left) and area under the curve (AUC) (right). (U) Representative liver histology by H&E staining (left) and Oil Red O staining (right). Scale bars = 50 µm. Each dot represents data from an individual biological replicate. Biological replicate numbers in each group are as follows: (figure 2B–H, O–U) *n* = 5, (figure 2I) *n* = 3, (figure 2J–L) *n* = 5–6, (figure 2M) *n* = 3–4. Data were shown as mean ± SEM. ^*^ *p* < 0.05, ^**^ *p* < 0.01, ^***^ *p* < 0.001 as determined by one‐way ANOVA followed by Tukey's multiple comparison (B–G), two‐tailed, unpaired Student's t‐test (J, M), or two‐way ANOVA followed by Tukey's multiple comparison (O–T).

To observe whether the gut microbiota influenced the *Card9* deficiency‐associated MASLD, we recolonized antibiotic‐treated HFD mice by fecal microbiome transplantation (FMT) using feces obtained from HFD‐fed WT mice or HFD‐fed *Card9* ^−^ mice. A control group received feces obtained from antibiotic‐treated HFD‐fed WT mice (Self‐FMT) (Figure 2A). We performed ITS sequencing on donor and recipient fecal samples. The ITS profiling demonstrated that the fungal community structure in recipient mice after FMT closely resembled that of the corresponding donors, supporting successful transfer and engraftment of donor‐associated mycobiota (Figure S2A,B). Our observations showed that body weight (Figure 2B), liver weight, and liver TG (Figure 2C) were increased in mice with FMT from HFD‐fed *Card9* ^−^ mice relative to HFD‐fed WT mice. The ALT and AST (Figure 2D) were significantly augmented in mice receiving FMT from HFD‐fed *Card9* ^−^ mice compared with those in mice receiving FMT from HFD‐fed WT mice, as well as the glucose concentration at fasting levels (Figure 2E). The mice administered FMT derived from HFD‐fed *Card9* ^−^ mice had higher blood glucose levels and impaired insulin sensitivity, as evidenced by the intraperitoneal glucose tolerance test (ipGTT) and the intraperitoneal insulin tolerance test (ipITT) (Figure 2F,G). Moreover, the TC, TG, and NEFA (Figure S2C) in serum were increased in mice treated with FMT derived from HFD‐fed *Card9* ^−^ mice than those treated with FMT derived from HFD‐fed WT mice. The histological analysis and Oil Red O staining of the liver (Figure 2H; Figure S2D) also showed profound steatosis in mice receiving FMT from HFD‐fed *Card9* ^−^ mice compared with those receiving FMT from HFD‐fed WT mice. Also, the proteins and genes involved in fatty acid oxidation, such as *Cpt1a*, *Mcad*, *Acat1*, and *Acox1*, were decreased in the livers of mice receiving FMT from HFD‐treated *Card9* ^−^ mice (Figure 2I; Figure S2E). However, the expression of genes involved in *de novo* lipogenesis, VLDL secretion, and FFAs transport showed no difference between mice administered FMT derived from HFD‐fed *Card9* ^−^ mice and those administered FMT derived from HFD‐fed WT mice (Figure S2F–H).

To further confirm whether the gut bacteria or gut mycobiota influenced the *Card9* deficiency‐associated MASLD, we analyzed the fecal contents of WT mice maintained on an HFD and *Card9* ^−^ mice maintained on an HFD by using bacterial 16S ribosomal DNA (16S rDNA) sequencing and fungal high‐throughput internal transcribed spacer (ITS) sequencing. Specifically, we observed that bacterial β‐diversity did not differ significantly between HFD‐fed WT mice and HFD‐fed *Card9* ^−^ mice (Figure S2I). Many bacterial taxa previously reported to be closely associated with MASLD showed no significant differences in relative abundance between HFD‐WT and HFD‐*Card9* ^−^ mice. These bacteria include *Coprococcus*, *Eubacterium*, *Lachnospiraceae*, *Acidaminococcus*, *Bifidobacterium*, and *Escherichia*, all of which are key taxa implicated in the regulation of hepatic steatosis and inflammatory responses (Figure S2J). These results demonstrated that the majority of MASLD‐related bacteria are not altered between HFD‐WT and HFD‐*Card9* ^−^ mice. Notably, there were two bacterial genera‐*Lactobacillus* and *Faecalibacterium*, that exhibited a decrease in the HFD‐*Card9* ^−^ group compared with the HFD‐WT group. The reduced abundance of *Lactobacillus* (a beneficial probiotic genus) and *Faecalibacterium* (a major butyrate‐producing, anti‐inflammatory genus) has been associated with the progression of MASLD in previous studies [35, 36]. However, in ITS sequencing, relative to WT mice, *Card9* ^−^ mice had decreased α‐diversity, indicated by Shannon, Simpson, and Chao1 after HFD treatment (Figure 2J). Principal component analysis (PCA) also revealed a significant alteration of gut mycobiota composition between HFD‐fed WT mice and HFD‐fed *Card9* ^−^ mice (Figure 2K). Specifically, we found the fungal microbiota *Saccharomyces cerevisiae* (*S. cerevisiae*) was notably diminished in HFD‐fed *Card9* ^−^ mice (Figure 2L). The result was further confirmed by detecting the burden of *S. cerevisiae* in the feces of mice (Figure 2M).

To further verify the impact of gut mycobiota during the development of *Card9* deficiency‐associated MASLD, we treated mice with antifungal Amphotericin B (AmB) during HFD for 12 weeks (Figure 2N). AmB treatment caused a substantial reduction in intestinal fungal burden, while bacterial 16S rDNA levels were slightly increased (Figure S2K). This effect was likely due to fungal clearance providing a more favorable niche for bacteria [10, 37]. However, the bacterial burden did not change between HFD‐WT and HFD‐*Card9* ^−^ mice. Interestingly, the differences between HFD‐fed *Card9* ^−^ mice and HFD‐fed WT mice mentioned above in body weight, liver weight, liver TG, serum ALT/AST, the glucose concentration at fasting levels, ipGTT, ipITT, histological analysis, and Oil Red O staining disappeared after AmB treatment (Figure 2O–U; Figure S2L). Additionally, the levels of serum TC, TG, and NEFA showed no significant variation (Figure S2M).

To further clarify the role of gut bacteria, we treated mice with a bacterial antibiotic cocktail (vancomycin, ampicillin, neomycin, and metronidazole) for 12 weeks of HFD feeding to directly assess whether the bacterial microbiota contributes to the development of *Card9* deficiency‐associated MASLD (Figure S2N). Our results showed that the MASLD phenotypic differences between HFD‐WT and HFD‐*Card9* ^−^ mice still persisted even under substantial bacterial depletion. These differences were supported by multiple parameters, including body weight, liver weight, hepatic TG content, serum ALT/AST levels, fasting glucose concentration, ipGTT, ipITT, serum TC, TG, and NEFA levels, as well as histological analysis and Oil Red O staining (Figure S2O–V). All these data demonstrated that *Card9* deficiency‐associated MASLD progression relies on gut mycobiota.

### 2.3 *S. cerevisiae* Alleviates *Card9* Deficiency‐Associated MASLD Progression

**FIGURE 3:**
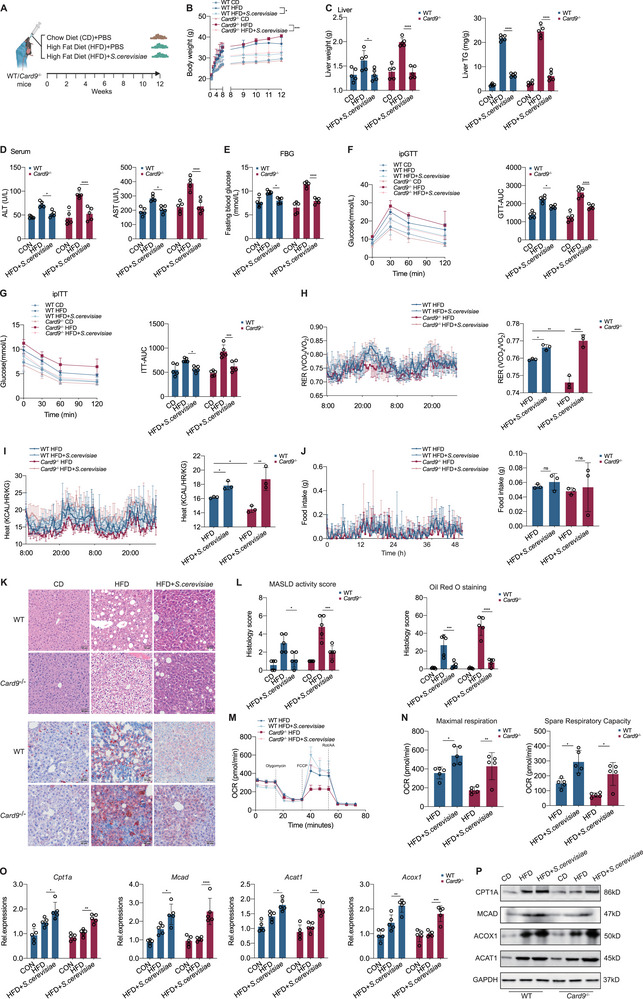
*S. cerevisiae* alleviates *Card9* deficiency‐associated MASLD progression. (A) Experimental schematic of WT and *Card9* ^−^ mice fed with Chow Diet (CD)/high‐fat diet (HFD) for 12 weeks. *S. cerevisiae* was gavaged to HFD mice every 3 days. (B) Body weight changes during the 12‐week experimental period. (C) Liver weight of mice in different groups (left) and content of Triglyceride (TG) in the liver of mice in different groups (right). (D) Levels of ALT and AST in serum. (E) The glucose concentration at fasting levels. (F) Levels of serum glucose for intraperitoneal glucose tolerance tests (ipGTT) (left) and area under the curve (AUC) (right). (G) Levels of serum glucose for intraperitoneal insulin tolerance test (ipITT) (left) and area under the curve (AUC) (right). (H, I) RER profiles and HEAT profiles of HFD‐fed WT mice and HFD‐fed *Card9* ^−^ mice with/without gavaging *S. cerevisiae*. (J) Food intake within 48 h. (K) Representative liver histology by H&E staining (up) and Oil Red O staining (down). Scale bars = 50µm. (L) MASLD activity score quantification and Oil Red O positive area quantification. (M) Oxygen consumption rate of hepatocytes from HFD‐fed WT mice and HFD‐fed *Card9* ^−^ mice with/without gavaging *S. cerevisiae* measured by Seahorse. (N) Individual parameters for maximum respiration and spare respiratory capacity were measured by Seahorse. (O) Q‐PCR analysis of relative mRNA expression levels of genes involved in fatty acid oxidation (*Cpt1a*, *Mcad*, *Acat1*, and *Acox1*) in mice livers. (P) Western blot analysis of relative protein involved in fatty acid oxidation (CPT1A, MCAD, ACOX1, ACAT1) in mouse livers. GAPDH was used as the internal standard. Each dot represents data from an individual biological replicate. Biological replicate numbers in each group are as follows: (figure 3B–G, K–P) *n* = 5, (figure 3H–J) *n* = 3. Data were shown as mean ± SEM. ^*^ *p* < 0.05, ^**^ *p* < 0.01, ^***^ *p* < 0.001 as determined by two‐way ANOVA followed by Tukey's multiple comparison (B–J, L, N, O).

*S. cerevisiae* has been proven to perform important functions in maintaining gut health [38, 39], such as interacting with the intestinal mucosa to improve intestinal‐related diseases. To assess the impact of *S. cerevisiae* on MASLD development, we evaluated *S. cerevisiae* colonization in HFD‐fed mice and found that it failed to stably colonize. *S. cerevisiae* was nearly undetectable in the intestine by day 4 after gavage (Figure S3A). Therefore, in vivo, HFD‐fed WT and HFD‐fed *Card9* ^−^ mice were gavaged with *S. cerevisiae* every 3 days for 12 weeks (Figure 3A). We found that the *S. cerevisiae* colonization group gained less body weight than the HFD group (Figure 3B), accompanied by increased fungal burden (Figure S3B). Liver weight and liver TG content were decreased in the *S. cerevisiae* colonization group (Figure 3C), along with serum ALT and AST levels as well as fasting glucose levels (Figure 3D,E). *S. cerevisiae* treatment reduced blood glucose levels and improved insulin sensitivity in HFD‐fed *Card9* ^−^ mice, as indicated by ipGTT and ipITT (Figure 3F,G). Moreover, the TC, TG, and NEFA (Figure S3C) in serum were all decreased by *S. cerevisiae* treatment. Correspondingly, in vivo metabolic cage also revealed that the HFD‐fed *Card9* ^−^ mice with *S. cerevisiae* treatment exhibited a higher respiratory exchange ratio (RER: VCO_2_/VO_2_), indicating improved metabolic flexibility, as well as increased heat production, with no changes in food intake (Figure 3H–J). Analysis of fecal TG output revealed that HFD led to elevated fecal TG excretion, whereas *S. cerevisiae* did not affect TG output (Figure S3D). The *S. cerevisiae* load also inhibited MASLD symptoms in HFD‐fed mice, as indicated by decreased MASLD activity score and hepatic steatosis (Figure 3K,L). Moreover, *S. cerevisiae* treatment reduced hepatic lipid accumulation in HFD‐mice (Figure S3E). To investigate how *S. cerevisiae* suppresses liver steatosis, we detected lipid metabolism in primary hepatocytes. The mitochondrial stress test showed *S. cerevisiae* colonization increased fatty acid oxidation, as indicated by upregulated maximal respiration and spare respiratory capacity (Figure 3M,N). The fatty acid oxidation in HFD‐fed *Card9* ^−^ mice was elevated with *S. cerevisiae* treatment as shown by upregulation of genes and proteins involved in fatty acid oxidation (Figure 3O,P), while the expression of genes involved in *de novo* lipogenesis, VLDL secretion, and FFAs transport was not changed with *S. cerevisiae* treatment (Figure S3F–H). Notably, heat‑killed S. cerevisiae (HK‐*S. cerevisiae*) retained a protective effect against HFD‑induced MASLD comparable to viable *S. cerevisiae*, as indicated by the body weight, liver weight, liver TG content, serum ALT/AST levels, fasting glucose concentration, ipGTT, ipITT, as well as histological analysis and Oil Red O staining (Figure S3I–Q). Accordingly, it is likely that components of *S. cerevisiae* that retain biological activity after heat inactivation contribute to the observed amelioration of MASLD. To further confirm the specific role of *S. cerevisiae* in *Card9* deficiency‐associated MASLD, we performed a fungal recolonization experiment by administering *S. cerevisiae* to both HFD‐WT and HFD‐*Card9* ^−^ mice by gavage after Amphotericin B treatment. Consistently, reconstitution with *S. cerevisiae* markedly attenuated the MASLD phenotype in Amphotericin B‐treated HFD‐WT and HFD‐*Card9* ^−^ mice (Figure S3R,S). In summary, these results suggested that *S. cerevisiae* alleviates *Card9* deficiency‐associated MASLD progression by regulating hepatic lipid homeostasis.

### 2.4 *S. cerevisiae* Induces Tryptophan Catabolites 5‐HIAA Production in the Intestine

**FIGURE 4:**
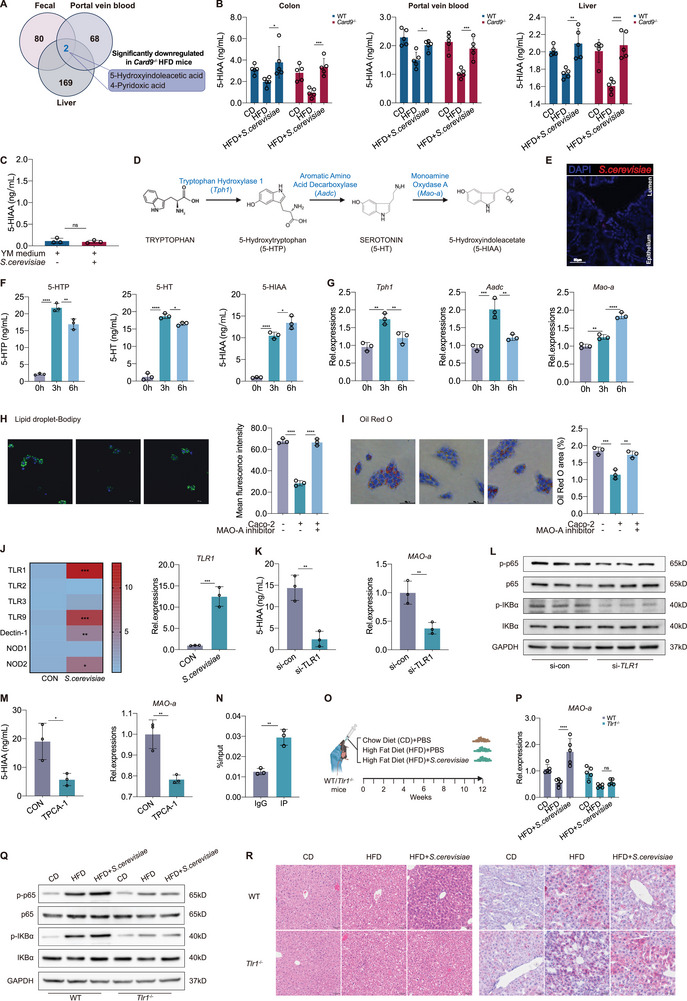
*S. cerevisiae* induces tryptophan catabolites 5‐HIAA production in the intestine via TLR1. (A) Venn diagram of all differentially metabolites between HFD‐fed WT and HFD‐fed *Card9* ^−^ mice in fecal, portal vein blood, and liver. (B) The content of 5‐HIAA in the colon, portal vein blood, and liver. (C) The content of 5‐HIAA in Yeast Malt Medium (YM) with/without *S. cerevisiae*. (D) The metabolic pathway of 5‐HIAA. (E) Representative images of fluorescently labeled *S. cerevisiae* (Cy5‐NHS ester, red) in the colon. (F) The content of 5‐HTP, 5‐HT, and 5‐HIAA with different time‐treatment. (G) Q‐PCR analysis of relative mRNA expression levels of *Tph1*, *Aadc*, and *Mao‐a* in Caco‐2 cells. (H) Bodipy staining for hepatocytes' lipid droplets (left). Quantification of mean fluorescence intensity (right). (I) Oil Red O staining for hepatocytes (left). Oil Red O positive area quantification (right). (J) Q‐PCR analysis of relative mRNA expression levels of fungal recognition receptor genes in Caco‐2 cells. (K) The content of 5‐HIAA in Caco‐2 cells with *TLR1* knockdown (left). Q‐PCR analysis of relative mRNA expression levels of *Mao‐a* in Caco‐2 cells with *TLR1* knockdown (right). (L) Western blot analysis of relative protein involved in NF‐κB pathway (p‐p65, p65, p‐IKBα, IKBα) in Caco‐2 cells. GAPDH was used as the internal standard. (M) The content of 5‐HIAA in Caco‐2 cells with TPCA‐1 treatment (left). Q‐PCR analysis of relative mRNA expression levels of *Mao‐a* in Caco‐2 cells with TPCA‐1 treatment (right). (N) ChIP with anti‐p65 of the regions containing the binding sites on the *Mao‐a* gene promoter in Caco‐2. (O) Experimental schematic of WT mice and *Tlr1^−^* mice fed with Chow Diet (CD)/high‐fat diet (HFD) for 12 weeks. *S. cerevisiae* was gavaged to HFD mice every 3 days. (P) Q‐PCR analysis of relative mRNA expression levels of *Mao‐a* in the mouse intestine. (Q) Western blot analysis of relative protein involved in NF‐κB pathway (p‐p65, p65, p‐IKBα, IKBα) in the mouse intestine. GAPDH was used as the internal standard. (R) Representative liver histology by H&E staining (left) and Oil Red O staining (right). Scale bars = 50 µm. Each dot represents data from an individual biological replicate. Biological replicate numbers in each group are as follows: (figure 4B, P–R) *n* = 5, (figure 4C, F–N) *n* = 3. Data were shown as mean ± SEM. ^*^ *p* < 0.05, ^**^ *p* < 0.01, ^***^ *p* < 0.001 as determined by two‐way ANOVA followed by Tukey's multiple comparison (B, P), two‐tailed, unpaired Student's t‐test (C, J, K, M, N), or one‐way ANOVA followed by Tukey's multiple comparison (F–I).

To investigate the mechanism of MASLD progression inhibited by *S. cerevisiae*, we compared the metabolites of feces, portal vein blood, and liver from WT‐HFD mice and *Card9* ^−^‐HFD mice using Untargeted metabolomic analysis (Figure S4A–C). We found 80 different metabolites in feces, 68 different metabolites in portal vein blood, and 134 different metabolites in liver between WT‐HFD mice and *Card9* ^−^‐HFD mice. Among these, only 5‐Hydroxyindoleacetic acid (5‐HIAA) and 4‐Pyridoxic acid were downregulated in feces, portal vein blood, and liver of HFD‐fed *Card9* ^−^ mice (Figure 4A), which may contribute to decreased hepatic steatosis through the “gut‐liver” axis. 5‐HIAA is a tryptophan metabolite predominantly produced in the intestine. In contrast, other tryptophan derivatives such as indoles, 2‐indolecarboxylic acid, indoleacrylic acid, and 3‐indoleacetonitrile exhibited no significant changes between WT‐HFD and *Card9* ^−^‐HFD mice, as evidenced by our liver untargeted metabolomic profiling (Figure S4D). To verify 5‐HIAA abundance by *S. cerevisiae* overload, we detected the content of 5‐HIAA in colon, portal vein blood, and liver in HFD‐fed WT and HFD‐fed *Card9* ^−^ mice gavaged with *S. cerevisiae*. The content of 5‐HIAA was all markedly increased under both viable *S. cerevisiae* and HK‐*S. cerevisiae* treatment (Figure 4B; Figure S4E), indicating that *S. cerevisiae* induces 5‐HIAA production.

Next, we sought to elucidate the possible mechanism by which *S. cerevisiae* enhances 5‐HIAA levels. First, we found that 5‐HIAA concentrations in culture supernatant remained unchanged during the growth period of *S. cerevisiae* with L‐Tryptophan addition, which indicated that 5‐HIAA is not a direct product of *S. cerevisiae* (Figure 4C). The production of 5‐HIAA is involved in the serotonin pathway, where tryptophan is catalyzed by Tryptophan Hydroxylase enzyme (TpH) and Aromatic Amino Acid Decarboxylase (AAAD) to produce Serotonin (5‐HT) and further 5‐HIAA [27] (Figure 4D). By gavaging *S. cerevisiae* into mice, we found that *S. cerevisiae* was in the vicinity of the intestinal epithelium (Figure 4E), pointing to the potential that *S. cerevisiae* might regulate 5‐HIAA production from intestinal epithelial cells. To verify whether intestinal epithelial cells secrete 5‐HIAA by *S. cerevisiae* treatment, we cultured intestinal epithelial Caco‐2 cells with *S. cerevisiae* and L‐Tryptophan. The production of 5‐HTP, 5‐HT, and 5‐HIAA was all elevated in Caco‐2 cells with *S. cerevisiae* treatment for 3 h, but only 5‐HIAA was increased until 6 h in the Caco‐2 cell supernatant after treatment with *S. cerevisiae* (Figure 4F). Although the expression of 5‐HTP production gene‐Tryptophan Hydroxylase 1 (*Tph1*), 5‐HT production gene‐Aromatic Amino Acid Decarboxylase (*Aadc*), and the 5‐HIAA production gene‐Monoamine Oxydase A (*Mao‐a*) were induced with 3h‐*S. cerevisiae* treatment, only the expression of *Mao‐a* was increased until 6h‐*S. cerevisiae* treatment (Figure 4G). We further cultured FFA‐treated primary hepatocytes with Caco‐2 and *S. cerevisiae*, in the case of adding L‐Tryptophan to the culture medium. By using MAO‐a‐specific antagonist clorgyline hydrochloride to inhibit 5‐HIAA production, the mRNA expression level of fatty acid oxidation genes (*Cpt1a*, *Mcad*, *Acat1*, *Acox1*) in primary hepatocytes was decreased (Figure S4F), thus lipid droplets in the hepatocytes increased (Figure 4H,I). To further investigate the regulation of 5‐HIAA production by *S. cerevisiae in vivo*, HFD‐WT mice were treated with a MAO‐a‐specific inhibitor, clorgyline hydrochloride, to inhibit 5‐HIAA production. We found that inhibition of MAO‐a abolished the protective effects of *S. cerevisiae* on MASLD, as indicated by the body weight, liver weight, liver TG content, serum ALT/AST levels, fasting glucose concentration, ipGTT, ipITT, as well as histological analysis and Oil Red O staining (Figure S4G–O). Next, we detected the receptors in Caco‐2 cells that can recognize fungi. Interestingly, Toll‐like receptor 1 (TLR1), Toll‐like receptor 9 (TLR9), Dendritic cell‐associated C‐type lectin‐1 (Dectin‐1), and Nucleotide‐binding oligomerization domain 2 (NOD2) were up‐regulated after *S. cerevisiae* treatment (Figure 4J; Figure S4P). However, only TLR1 regulated the production of 5‐HIAA and the expression of *Mao‐a*, whereas TLR9, Dectin‐1, and NOD2 exhibited no such effects (Figure 4K; Figure S4Q). TLR1, as a pattern recognition receptor (PRR), can respond to pathogen‐associated molecular patterns (PAMPs) and activate the downstream myeloid differentiation primary response 88‐nuclear factor κB (MyD88‐NF‐κB) signaling pathway [40, 41]. We found that TLR1 knockdown attenuated NF‐κB phosphorylation induced by *S. cerevisiae* treatment in Caco‐2 cells (Figure 4L). Furthermore, we found that in the presence of TPCA‐1 (a selective inhibitor of IKKβ/NF‐κB signaling), *S. cerevisiae* failed to promote the production of 5‐HIAA and the expression of *Mao‐a* (Figure 4M). Subsequent chromatin immunoprecipitation (ChIP) assays in Caco‐2 cells confirmed that NF‐κB p65 subunit directly binds to the *Mao‐a* promoter region upon *S. cerevisiae* stimulation (with IgG as essential internal controls) (Figure 4N). To further explore the protective effect of *S. cerevisiae* against MASLD via TLR1 in vivo, WT and *Tlr1* ^−^ mice were fed a HFD for 12 weeks, with *S. cerevisiae* administered via gavage throughout the feeding period (Figure 4O). Consistent with our in vitro findings, *S. cerevisiae* failed to induce *Mao‐a* expression or activate the NF‐κB signaling pathway in *Tlr1^−^* mice intestine (Figure 4P,Q), and the protective effect of *S. cerevisiae* on MASLD was abolished, as indicated by the body weight, liver weight, liver TG content, serum ALT/AST levels, fasting glucose concentration, ipGTT, ipITT, as well as histological analysis and Oil Red O staining (Figure 4R; Figure S5A–I). The data above demonstrated a novel “microbe‐host” axis in which *S. cerevisiae* affects the specific tryptophan catabolites 5‐HIAA production in the intestine through TLR1 to inhibit hepatic lipid deposition.

### 2.5 5‐HIAA Ameliorates *Card9* Deficiency‐Associated MASLD by Enhancing Fatty Acid Oxidation

**FIGURE 5:**
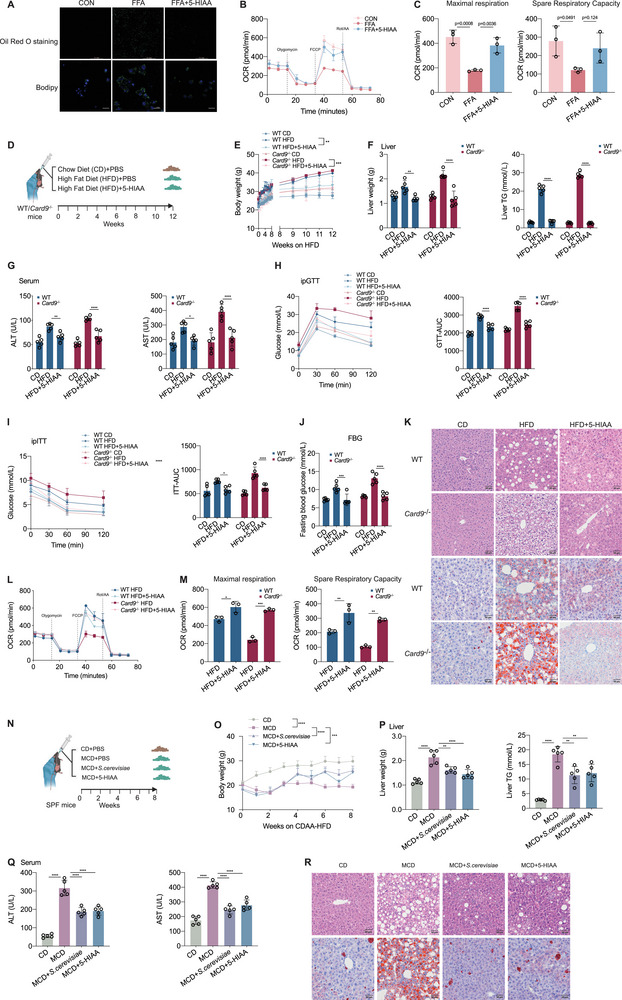
5‐HIAA ameliorates *Card9* deficiency‐associated MASLD by enhancing fatty acid oxidation. (A) Oil Red O staining (upper) and bodipy staining (down). (B) Oxygen consumption rate of hepatocytes treated with 5‐HIAA measured by Seahorse. (C) Individual parameters for maximum respiration and spare respiratory capacity were measured by Seahorse. (D) Experimental schematic of WT and *Card9* ^−^ mice fed with Chow Diet (CD)/high‐fat diet (HFD) for 12 weeks. 5‐HIAA was administered to HFD mice every 3 days. (E) Body weight changes during the 12‐week experimental period. (F) Liver weight of mice in different groups (left) and content of Triglyceride (TG) in the liver of mice in different groups (right). (G) Levels of ALT and AST in serum. (H) Levels of serum glucose for intraperitoneal glucose tolerance tests (ipGTT) (left) and area under the curve (AUC) (right). (I) Levels of serum glucose for intraperitoneal insulin tolerance tests (ipITT) (left) and area under the curve (AUC) (right). (J) The glucose concentration at fasting levels. (K) Representative liver histology by H&E staining (upper) and Oil Red O staining (lower). Scale bars = 50µm. (L) Oxygen consumption rate of hepatocytes from HFD‐fed WT mice and HFD‐fed *Card9* ^−^ mice with/without gaveging 5‐HIAA measured by Seahorse. (M) Individual parameters for maximum respiration and spare respiratory capacity were measured by Seahorse. (N) Experimental schematic of WT mice fed with Chow diet (CD)/methionine–choline‐deficient (MCD) diet for 8 weeks. *S.crervisiae*/5‐HIAA was gavaged to MCD mice every 3 days. (O) Body weight changes during the 8‐week experimental period. (P) Liver weight of mice in different groups (left) and content of Triglyceride (TG) in the liver of mice in different groups (right). (Q) Levels of ALT and AST in serum. (R) Representative liver histology by H&E staining and Oil Red O staining. Scale bars = 50 µm. Each dot represents data from an individual biological replicate. Biological replicate numbers in each group are as follows: (figure 5A–C, L, M) *n* = 3, (figure 5E–K, O–R) *n* = 5. Data were shown as mean ± SEM. ^*^ *p* < 0.05, ^**^ *p* < 0.01, ^***^ *p* < 0.001 as determined by one‐way ANOVA followed by Tukey's multiple comparison (C, O–Q) or two‐way ANOVA followed by Tukey's multiple comparison (E–M).

To explore the role of 5‐HIAA in liver lipid deposition, we first assessed the effect of 5‐HIAA on hepatocytes treated with FFAs [42]. FFAs treatment increased the content of TG in hepatocytes at 36h and 48h compared with no FFAs treatment hepatocytes (Figure S6A). Dose‐dependent 5‐HIAA treatment had no significant impact on cell viability (Figure S6B), and the content of TG was reduced following 48 h of 5‐HIAA treatment in a dose‐responsive manner (Figure S6C). Lipid droplet assessment via Flow cytometry demonstrated a significant decrease in hepatocyte TG accumulation upon 5‐HIAA treatment (Figure S6D). Consistent results were also observed in Oil Red O (ORO) staining and bodipy staining (Figure 5A; Figure S6E). Particularly, the fatty acid oxidation was increased as indicated by upregulated oxygen consumption rate (OCR) (Figure 5B), including increased maximal respiration and spare respiratory capacity (Figure 5C). And fatty acid oxidation‐related protein and genes (*Cpt1a, Mcad*, *Acat1*, *Acox1*) in hepatocytes were significantly increased under 5‐HIAA treatment (Figure S6F,G). However, gene expression related to *de novo* lipogenesis, VLDL secretion, and FFA transport showed no appreciable alterations (Figure S6H–J).

To further clarify whether 5‐HIAA participates in the *Card9*deficiency‐associated MASLD progression in vivo, we administered HFD‐fed WT mice and HFD‐fed *Card9* ^−^ mice by gavaging 5‐HIAA for 12 weeks (Figure 5D). The 5‐HIAA treatment group showed a higher level of 5‐HIAA in the liver contents (Figure S6K), indicating that 5‐HIAA may exert greatly systemic microbiota‐regulated effects. We found that the 5‐HIAA‐treated group exhibited  lower body weight, liver weight, and liver TG content (Figure 5E,F), along with reduced serum ALT and AST levels (Figure 5G). The insulin sensitivity had been improved by 5‐HIAA treatment, as indicated by the intraperitoneal glucose tolerance test (ipGTT) and the intraperitoneal insulin tolerance test (ipITT) (Figure 5H,I). Furthermore, the glucose concentration at fasting levels was lower in the 5‐HIAA‐treated group (Figure 5J). The TC, TG, and NEFA (Figure S6L) in serum were also decreased after 5‐HIAA treatment. Also, 5‐HIAA alleviated MASLD progression in HFD‐fed mice (Figure 5K; Figure S6M). Moreover, 5‐HIAA treatment reduced hepatic lipid accumulation in HFD‐mice (Figure S6N). Besides, the cellular oxygen consumption rate, including maximal respiration and spare respiratory capacity, was increased after 5‐HIAA treatment (Figure 5L,M). In addition, the levels of genes and proteins associated with fatty acid oxidation were upregulated in the 5‐HIAA treatment group (Figure S6O,P) without affecting genes involved in *de novo* lipogenesis, VLDL secretion, and FFAs transport (Figure S6Q–S). These results revealed that 5‐HIAA ameliorates hepatic steatosis associated with *Card9* knockout via upregulating fatty acid oxidation.

Given the therapeutic effect of *S. cerevisiae* and 5‐HIAA on MASLD, we aimed to further validate their potential role in MASLD/MASH. We orally gavaged *S. cerevisiae* and 5‐HIAA into WT male mice fed a methionine–choline‐deficient (MCD) diet within 8 weeks to explore the effect of *S. cerevisiae* and 5‐HIAA on MASLD/MASH progression (Figure 5N). In the MCD diet model, we found that body weight (Figure 5O), liver weight and hepatic TG levels (Figure 5P), as well as plasma ALT and AST levels (Figure 5Q) of the *S. cerevisiae* and 5‐HIAA‐gavaged mice were lower than those of the MCD‐fed mice. By hematoxylin and eosin (H&E) and Oil Red O staining (Figure 5R), we found that *S. cerevisiae* and 5‐HIAA‐gavaged mice showed improvements in MASLD/MASH progression, with a lower MASH activity score and reduced lipid droplet accumulation compared with MCD‐fed mice (Figure S6T). These findings indicate that *S. cerevisiae* and 5‐HIAA are promising therapeutic approaches against MASLD/MASH.

### 2.6 5‐HIAA Triggers CPT1A/ACOX1 Transactivation by AhR Nuclear Translocation

**FIGURE 6:**
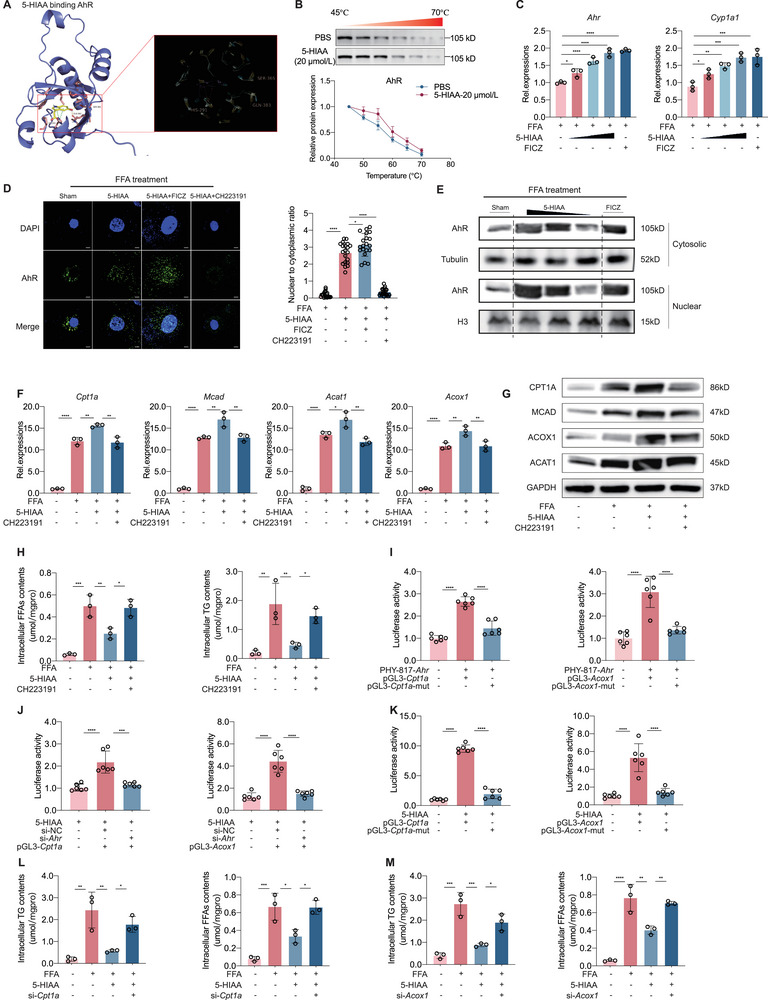
5‐HIAA triggers *Cpt1a* and *Acox1* transactivation by AhR to alleviate hepatic steatosis. (A) The docking model shows the predicted binding mode of 5‐HIAA to AhR. (B) Cellular thermal shift assay between AhR and 5‐HIAA. (C) Q‐PCR analysis of relative mRNA expression levels of *Ahr* and *Cyp1a1* in AML12. The concentration of 5‐HIAA is 62.5, 125, and 250 µm/L. (D) Immunofluorescence analysis of AhR in hepatocytes. Scale bar = 5 µm. Quantification of nuclear versus cytoplasmic fluorescence intensity. Individual data points correspond to measurements from single cells. A total of 20 morphologically intact hepatocytes were randomly selected for each experimental group, which were derived from three independent biological replicates. (E) Western blot analysis of AhR in the cytoplasm and the nucleus. Tublin was used as the cytoplasm standard. H3 was used as the nuclear standard. (F) Q‐PCR analysis of relative mRNA expression levels of genes involved in fatty acid oxidation (*Cpt1a*, *Mcad*, *Acat1*, *Acox1*) in hepatocytes. (G) Western blot analysis of relative protein involved in fatty acid oxidation (CPT1A, MCAD, ACAT1, ACOX1) in hepatocytes. GAPDH was used as the internal standard. (H) The content of intracellular FFAs and TG in hepatocytes. (I–K) The relative luciferase activity was calculated by dividing the firefly luciferase activity by the Renilla luciferase activity. (L, M) The content of intracellular TG and FFAs in AML12. Data were shown as mean ± SEM. Each dot represents data from an individual biological replicate. Biological replicate numbers in each group are as follows: (figure 6B–I, N, O) *n* = 3, (figure 6J–M) *n* = 6. Data were shown as mean ± SEM. ^*^ *p* < 0.05, ^**^ *p* < 0.01, ^***^ *p* < 0.001 as determined by one‐way ANOVA followed by Tukey's multiple comparison (C, D, F, H–O).

We further explored the molecular mechanism mediating FAO upregulation by 5‐HIAA stimulation. 5‐HIAA is a ligand for the AhR [43], which was also shown by molecular docking (Figure 6A). Additionally, we performed a cellular thermal shift assay to investigate the binding affinity between 5‐HIAA and AhR. The results showed that 5‐HIAA treatment increased the thermal stability of AhR protein, reflected in the slower shift of protein thermal melting curves compared with PBS‐treated (Figure 6B). Then, we used FICZ as an AhR agonist and CH223191 as an AhR antagonist to investigate 5‐HIAA function. The results showed that 5‐HIAA promoted the expression of *Ahr* and *Cyp1a1* (target gene of AhR) (Figure 6C). As AhR is reported to translocate to the nucleus, we detected AhR location upon 5‐HIAA treatment. The result showed that 5‐HIAA promoted AhR transfer to the nucleus, which was promoted by FICZ but blocked by CH223191 (Figure 6D,E). Obviously, the administration of the AhR‐specific antagonist CH223191 led to a decrease in FAO induced by 5‐HIAA (Figure 6F,G). Furthermore, the reduction of intracellular FFAs and intracellular TG caused by 5‐HIAA was also reversed by CH223191 (Figure 6H). These findings suggested that 5‐HIAA promotes FAO through AhR translocation to the nucleus and upregulation of AhR expression.

It has been reported that AhR binds to FAO genes‐*Cpt1a* and *Acox1* [44]. Consistent with this, we overexpressed AhR and co‐transfected pGL3‐*Cpt1α*/pGL3‐*Acox1* reporter plasmid or pGL3‐*Cpt1α*‐mut/pGL3‐*Acox1*‐mut reporter plasmid in HEK293T cells. AhR overexpression significantly increased luciferase activity driven by the wild‐type promoters, whereas this effect was markedly attenuated when the putative AhR binding sites were mutated (Figure 6I). Next, we assessed the functional effects of 5‐HIAA on *Cpt1a* and *Acox1* transactivated by AhR. The expression of *Cpt1a*/*Acox1* increased dramatically with 5‐HIAA treatment in AML12 cells, while their expression was decreased by *si‐Ahr* treatment (Figure 6J). Furthermore, AML12 cells were transfected with either wild‐type or mutant *Cpt1α*/*Acox1* promoter reporters and treated with 5‐HIAA. Consistently, 5‐HIAA treatment enhanced luciferase activity of the wild‐type promoters, whereas this response was largely abolished in the mutant constructs (Figure 6K). Finally, the 5‐HIAA‐induced reductions in intracellular FFAs and TG were also abrogated by *si‐Cpt1a* and *si‐Acox1* treatment (Figure 6L,M). These results demonstrated that AhR transactivates *Cpt1a* and *Acox1* to promote FAO under 5‐HIAA treatment.

### 2.7 *S. cerevisiae* Supplement Effectively Ameliorates Hepatic Steatosis in MASLD Patients

**FIGURE 7:**
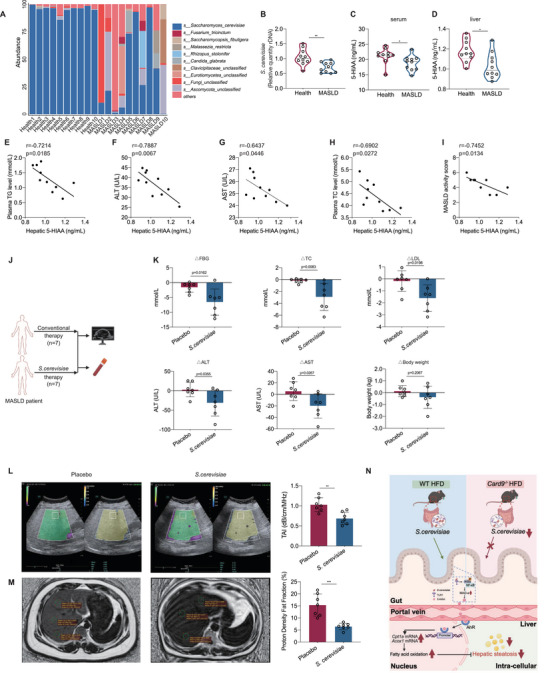
*S. cerevisiae* supplement effectively ameliorated hepatic steatosis in MASLD patients. (A) Barplot analysis of the species level of the gut fungal community in the feces of healthy individuals and MASLD patients. (B) The burden of *S. cerevisiae* in the feces of healthy individuals and MASLD patients. (C, D) The content of 5‐HIAA in serum and livers of healthy individuals and MASLD patients. Pearson r and p values for relative hepatic 5‐HIAA levels with plasma TG level (E), plasma ALT level (F), plasma AST level (G), plasma TC level (H), and MASLD activity score (I) of MASLD patients (*n* = 10). (J) Study design for patients with MASLD who received Placebo/*S. cerevisiae* therapy. (K) Effects of *S. cerevisiae* on fasting blood glucose (FBG), total cholesterol (TC), low‐density lipoprotein (LDL), alanine aminotransferase (ALT), and aspartate aminotransferase (AST) in serum, and on body weight in patients with MASLD. △MASLD parameter = MASLD parameter after treatment‐MASLD parameter at baseline. (L) Representative images of ultrasound attenuation analysis (USAT) in MASLD patients with Placebo and *S. cerevisiae* therapy. Distribution of tissue attenuation imaging (TAI) values. (M) Liver fat content measurement with MRI‐based proton density fat fraction (PDFF) in MASLD patients with Placebo and *S. cerevisiae* therapy. Distribution of proton density fat fraction (PDFF) values. (N) Overview schematic of the study. The dots show individual participant measurements. Participant numbers in each group are as follows: (figure 7A–I) *n* = 10, (figure 7K–M) *n* = 7. Data were shown as mean ± SEM. ^*^ *p* < 0.05, ^**^ *p* < 0.01, ^***^ *p* < 0.001 as determined by two‐tailed, unpaired Student's t‐test (B–D, K–M). Correlation significance was determined by using linear regression (E–I).

To validate our findings in the clinic, we first collected feces from MASLD patients for ITS sequencing. Principal Component Analysis (PCA) demonstrated significant differences in  fungal communities among healthy individuals and MASLD patients (Figure S7A). To investigate alterations in gut mycobiota composition, we assessed the relative proportions of major taxa across the different groups. At the genus level, *Saccharomyces* showed a reduction in MASLD patients (Figure S7B). On the species scale, *S. cerevisiae* was significantly reduced in MASLD patients, and the detection of *S. cerevisiae* burden in feces also verified this result (Figure 7A,B; Figure S7C). We also measured 5‐HIAA levels in serum and livers and found they were decreased in MASLD patients compared with healthy individuals (Figure 7C,D). Furthermore, correlation analysis revealed that 5‐HIAA content of livers was negatively correlated with plasma TG, ALT, AST, TC levels, and MASLD activity score (Figure 7E–I). In conclusion, *S. cerevisiae* and 5‐HIAA were negatively correlated with the clinical MASLD phenotypes. Additionally, we conducted a randomized, double‐blind clinical trial to explore the effect of *S. cerevisiae* in MASLD patients (Figure 7J), with endpoints including serum biomarkers such as fasting blood glucose (FBG), total cholesterol (TC), low‐density lipoprotein (LDL), alanine aminotransferase (ALT), aspartate aminotransferase (AST), and liver fat content. The baseline characteristics between the Placebo group and the *S. cerevisiae* group were comparable and were summarized in Table S4. Compared with Placebo therapy, the patients with MASLD who received *S. cerevisiae* treatment for 8 weeks showed a reduction in serum FBG, TC, LDL, ALT, and AST, together with a trend toward reduced body weight (Figure 7K). Consistent with this result, a reduction of liver fat content after *S. cerevisiae* treatment was observed on ultrasound attenuation analysis (USAT) and magnetic resonance imaging‐proton density fat fraction(MRI‐PDFF) (Figure 7L,M). In summary, the results showed gut fungi‐*S. cerevisiae* stimulated intestinal 5‐HIAA production to alleviate MASLD progression by enhancing fatty acid oxidation (Figure 7N), highlighting that the mycobiota‐dependent gut‐liver axis was a promising target for the prevention of MASLD.

## 3 Discussion

MASLD is recognized as one of the most common causes of chronic liver disease worldwide, with its incidence increasing steadily across the globe each year [45]. The molecular mechanisms underlying MASLD are highly intricate and have not been fully clarified. According to current consensus, the progression of MASLD involves a complex interplay of multiple factors, such as free fatty acids (FFAs) absorption, *de novo* lipogenesis, fatty acid oxidation, and hepatic lipid export [29, 30, 32, 34]. Diminished fatty acid oxidation is considered a major culprit underlying the accumulation of toxic lipid species [46]. However, fatty acid oxidation (FAO) is recognized as a dynamic process during the progression of MASLD [47]. Enhanced oxidative metabolism has been documented in multiple obese models of MASLD [48, 49, 50], potentially to relieve ER stress and lipoapoptosis. Furthermore, clinical studies have reported increased mitochondrial oxidation in the liver of patients with MASLD [50]. These data suggest the operation of a mechanism of “hepatic metabolic flexibility”, by which liver mitochondria adapt to altered bioenergetic demands preceding advanced MASLD [51]. Fungal variations can influence the host metabolism, but the role of gut mycobiota in MASLD progression remains unclear. In the present research, we observe that *Card9* ^−^ mice display a more serious illness of MASLD under HFD treatment. *Card9* deficiency led to disturbance of intestinal fungi, especially decreased *S. cerevisiae*. *S. cerevisiae* induced the production of tryptophan metabolite 5‐HIAA in the intestine through TLR1, eventually mitigating liver steatosis. Mechanistically, 5‐HIAA activates “AhR‐CPT1A/ACOX1 axis”‐mediated fatty acid oxidation, resulting in decreased hepatic TG accumulation. Thus, our study reveals the role of commensal fungi in the gut‐liver axis during the development of MASLD.

Recently, the influence of commensal microbes in maintaining host homeostasis has garnered considerable interest. Strong evidence indicates that gut microbes have been linked to the development of obesity [52], T2D [53], and even cancer [54]. As to the regulation of the whole‐body lipid metabolism, commensal bacteria, such as *Bifidobacterium bifidum* V and *Lactobacillus plantarum* X, significantly reduced the development of MASLD [55]. Furthermore, *Bacteroides xylanisolvens* was reported to reduce hepatic steatosis [56]. Regarding commensal fungi, *C. albicans* represents the main yeast species residing in the human intestine [57]. Our earlier research demonstrated that imbalances in commensal fungi can contribute to the progression of colitis‐associated colon cancer (CAC) [23]. Although recent work has reported that intestinal filamentous fungi‐*Fusarium* spp reversed MASH progression through a secondary metabolite [18], the role of commensal fungi in MASLD clinical treatment is elusive. In this work, we reveal the significant contribution of commensal fungi to MASLD progression. By using fungal high‐throughput internal transcribed spacer (ITS) sequencing with feces from HFD‐fed *Card9* ^−^ mice, antifungal Amphotericin B‐treated HFD‐fed mouse model, and broad‐spectrum antibacterial antibiotic‐treated HFD‐fed mouse model, we present strong evidence that disruptions in commensal fungi can promote the development of MASLD. However, it must be acknowledged that antibiotics can exert potential off‐target effects. In addition to reducing microbial burden, antibiotics may exert direct effects on host physiology, leading to off‐target effects. To address this issue, we performed a fungal recolonization experiment, demonstrating that the observed phenotypic changes were mediated by the depletion of gut fungi rather than off‐target effects. Of note, we conducted a preliminary clinical trial and identified that *S. cerevisiae* reduces hepatic steatosis in MASLD patients, providing a promising therapeutic approach against MASLD.

*S. cerevisiae* is a well‐studied natural colonizer of the human gut [58], exerting the potential application and a well‐tolerated safety profile to improve irritable bowel syndrome (IBS) [59, 60]. The beneficial effects of *S. cerevisiae* can be attributed to its activity to release various saccharolytic enzymes that can facilitate the generation of short‐chain fatty acids (SCFAs) and alcohols by gut microbial communities, compounds recognized for their prokinetic effects, particularly in the small intestine [61, 62]. *S. cerevisiae* was also found to mitigate inflammation and suppress colorectal tumorigenesis in AOM/DSS‐induced CRC mouse models by strengthening the intestinal barrier, reducing levels of proinflammatory cytokines and markers of tumor cell proliferation, and elevating SCFA concentrations in fecal matter [63].

Our findings also provide an improved insight into the understanding of the intestinal Trp metabolism caused by fungal colonization and its role in liver metabolism remodeling. Trp metabolism is affected under many pathological conditions, such as inflammatory bowel diseases [64, 65, 66, 67, 68], metabolic syndrome and obesity [69, 70, 71], and infectious diseases [71, 72, 73]. The intestinal microbiota is an important participant in intestinal Trp metabolism [74]. *Corynebacterium* spp., *Streptococcus* spp., and *Escherichia coli* are reported to synthesize serotonin in culture [75]. But the mechanism by which intestinal fungi affect Trp metabolism is still unclear. Here, LC–MS/MS‐based untargeted metabolomics analysis reveals that 5‐HIAA, a catabolite of the Trp metabolic pathway affected by *S. cerevisiae*, is lower in MASLD. Analyses conducted both in vitro and in vivo indicate that 5‐HIAA may serve as a promising treatment for MASLD. Although the precise role of 5‐HIAA in the liver immune microenvironment is unknown, growing evidence suggests that 5‐HIAA has profound immunomodulatory effects on various immune cells. 5‐HIAA is reported to be a ligand for GPR35 to promote neutrophil recruitment to sites of inflammation to eliminate bacteria [76]. Another study reveals that 5‐HIAA suppresses arthritis by activating AhR in Breg cells [43]. Furthermore, recent studies have shown that bacteria directly produce 5‐HIAA, which improves type 2 diabetes (T2D), indicating that 5‐HIAA is of great significance for metabolism‐related diseases [28]. In our work, we find *S. cerevisiae* does not directly produce 5‐HIAA, but influences intestinal 5‐HIAA production. Furthermore, the molecular mechanism by which *S. cerevisiae* selectively increases 5‐HIAA in the intestine, along with the difference between host‐derived and microbe‐derived 5‐HIAA in the context of metabolic disease, requires further exploration.

In conclusion, our study reveals the influence and impact of commensal mycobiota in the regulation of MASLD progression through the “gut‐liver axis” and initially suggests *S. cerevisiae* and a catabolite of the Trp metabolic pathway, 5‐HIAA, as a novel strategy of therapy for restoring lipid homeostasis in the liver. Given the notable effects of 5‐HIAA and the beneficial effects of *S. cerevisiae* on MASLD relief, it is possible that, beyond MASLD, *S. cerevisiae* and 5‐HIAA could be used as prebiotics to ameliorate other metabolic diseases.

However, this study has several limitations. First, the *Card9* ^−^ mice used in this study represent a global knockout model. Given that CARD9 is broadly expressed in myeloid cells and participates in multiple innate immune signaling pathways, we cannot fully exclude the possibility that systemic immune alterations contribute to the observed metabolic phenotype. Specifically, mechanisms beyond the proposed mycobiota‐5‐HIAA‐AhR axis, such as impaired inflammasome activation, might be involved. Since CARD9 is critical for intestinal inflammasome activation [77]—a pathway known to protect against hepatic steatosis [78]—its deficiency in our global knockout model might partially contribute to the exacerbated MASLD. Although our data firmly link *Card9* deficiency to the altered *S. cerevisiae*‐5‐HIAA axis and impaired hepatic lipid metabolism, future studies using tissue‐ or cell type‐specific *Card9* conditional knockout models are required to definitively distinguish the precise contributions of intestinal versus systemic myeloid compartments. Additionally, our results indicate that treatment with *S. cerevisiae* increases systemic energy metabolism in mice, although this finding still requires further validation, such as measurements of iBAT, eWAT, iWAT, muscle weight, and thermogenic activity of BAT and WAT [79]. Second, while our in vitro data using Caco‐2 cells suggest a potential role for TLR1 in mediating the effects of *S. cerevisiae*, it is important to acknowledge the inherent limitations of this cancer‐derived cell line, which may not fully recapitulate the complex structure, differentiation status, and functional behavior of primary intestinal epithelial cells in vivo. Therefore, the physiological relevance of these in vitro findings requires further validation using more physiologically relevant models, such as primary intestinal epithelial cells or primary intestinal organoids, in future studies. Third, the receptor specificity of 5‐HIAA requires further investigation. Notably, in addition to AhR, 5‐HIAA has been identified as a ligand for other receptors, most notably GPR35. For example, 5‐HIAA has been reported to bind GPR35 expressed on both neutrophils and eosinophils, thereby promoting their recruitment to sites of inflammation [76, 80]. Furthermore, considering the complexity of tryptophan metabolite signaling, we cannot rule out the possibility that 5‐HIAA might interact with other metabolite‐sensing receptors, such as the hydroxycarboxylic acid receptor 2 (HCA2, a known receptor for the tryptophan‐derived metabolite), or the pregnane X receptor (PXR, a well‐established receptor for the microbial tryptophan metabolite). Although our data indicate that 5‐HIAA promotes AhR nuclear translocation and activates the AhR‐CPT1A/ACOX1 axis to enhance fatty acid oxidation, these findings support the involvement of AhR but do not definitively establish it as the sole mediator of 5‐HIAA action. Therefore, we cannot exclude the possibility that additional receptors contribute to the metabolic effects of 5‐HIAA in MASLD. Future studies using receptor‐specific genetic models or pharmacological inhibitors, such as GPR35 antagonists, will be needed to further define the relative contribution of these pathways. Fourth, the clinical study has several important limitations. Although this study included a placebo‐controlled design (Placebo, *n* = 7; *S. cerevisiae*, *n* = 7), the overall sample size remains relatively small, which may limit the statistical power of the analysis. In addition, the intervention duration was relatively short (8 weeks), which may not fully capture the long‐term metabolic effects of *S. cerevisiae*. Therefore, larger‐scale, randomized, placebo‐controlled trials with extended follow‐up periods will be required to further validate the therapeutic potential of *S. cerevisiae* and its regulation of 5‐HIAA in MASLD patients. Finally, from a microbiological perspective, our study utilized a single strain of *S. cerevisiae* to elucidate its protective role. It is well established that different *S. cerevisiae* strains vary dramatically in their metabolic capabilities and functional profiles [81, 82]. For instance, the highly domesticated laboratory reference strain S288C used herein offers a standardized genetic and cell wall background, making it an ideal model for pinpointing specific host‐receptor interactions. *S. cerevisiae* var. *boulardi*, a widely utilized probiotic strains, possess distinct evolutionary adaptations, including enhanced thermotolerance, robust resistance to gastric acid and bile salts, and increased adhesive capacity. Consequently, future studies comparing a diverse panel of *S. cerevisiae* strains are warranted to determine whether the beneficial effects observed here can be broadly extended. Moreover, our results demonstrate that heat‐inactivated *S. cerevisiae* still increased 5‐HIAA levels and ameliorated MASLD‐related phenotypes, suggesting that heat‐stable components of the yeast cell wall (β‐glucans, α‐mannan, mannoproteins, and chitin) may exert protective effects by binding to host receptors [83, 84, 85] or luminal metabolites [86, 87], thereby improving MASLD. Future studies are needed to identify the specific microbial factors responsible for this hepatoprotection. In addition, the restoration of metabolic flexibility reflected by the increased RER [88] following *S. cerevisiae* treatment suggests that this yeast may alleviate MASLD through extrahepatic mechanisms, in addition to enhancing hepatic fatty acid oxidation. Furthermore, our study provides only preliminary evidence for the beneficial effects of *S. cerevisiae* against MASH; its effects on hepatic inflammatory markers and precise mechanisms underlying its anti‐inflammatory action remain to be fully elucidated.

## 4 Material and Methods

### 4.1 Patients and Specimens

To explore the fungal composition in the feces of patients with MASLD, and the correlation between 5‐HIAA and MASLD, we collected 10 feces samples, 10 serum samples, and 10 liver samples from MASLD patients, and 10 controls were obtained. The above‐mentioned samples were all derived from Nanjing Drum Tower Hospital. All collected samples were evaluated by trained pathologists who verified the disease diagnoses. Patient clinical characteristics are summarized in Table S1. Before participation, all individuals provided prior information and written informed consent. The use of these patient specimens was authorized by the Ethics Committee of Nanjing Drum Tower Hospital (ChiCTR2400094716).

To test the potential effects of *S. cerevisiae* in the treatment of MASLD, we recruited fourteen individuals with MASLD from the Hospital of Integrated Traditional Chinese and Western Medicine, Nanjing University of Chinese Medicine. The clinical information of these patients is listed in Table S2. These 14 patients were randomly divided into the placebo group and the *S. cerevisiae* group. Participants in the *S. cerevisiae* group received oral dried yeast tablets (manufactured by Guangdong Wuzhou Pharmaceutical Co., Ltd.). Each 0.2 g tablet contained 1 × 10^9^ CFU of viable *S. cerevisiae*. Participants took 0.6 g (three tablets) per administration, three times daily, for 8 consecutive weeks. For the placebo group, participants received orally administered placebo tablets for 8 consecutive weeks at the same dosage regimen: three tablets per administration, three times daily. The placebo tablets consisted of inert pharmaceutical excipients without any active probiotic component. Notably, the *S. cerevisiae* tablets and placebo tablets were identical in terms of color, odor, appearance, and packaging, making them completely indistinguishable to participants. Lifestyle instruction was provided by the investigators, with a relatively stable diet and continued exercise recommended for the duration of the intervention. The *S. cerevisiae* or the placebo was administered on the background of a stable diet and exercise regimen.

At baseline and after 8 weeks of therapy, peripheral blood samples were collected and centrifuged at 1000 × g and 4°C for 5 min to obtain the serum. The levels of blood biochemical indicators (i.e., FBG, TC, LDL, TG, AST, and ALT) were measured using an autoanalyzer (BioTek Instruments 800TS). Prior information and written consent were obtained from all participants before starting our project. This study of patient specimens was approved by the Ethics Committee of the Hospital of Integrated Traditional Chinese and Western Medicine, Nanjing University of Chinese Medicine (2025‐LWKY‐020).

### 4.2 Mice Experiments

All animal procedures were conducted in strict compliance with the NIH Guide for “the Care and Use of Laboratory Animals”, and were evaluated and sanctioned by the Institutional Animal Care and Use Committee of the Medical College of Nanjing University (approval number: IACUC‐D2202102). Mice were maintained in specific pathogen‐free conditions within a temperature range of 20°C–26°C and relative humidity conditions of 40%–70%, maintained on a 12‐h light/dark regimen. Food and water were available at all times. Dr. Xin Lin (Tsinghua University, Beijing, China) supplied the *Card9* ^−^ mice used in this study, and they were employed following nine generations of backcrossing onto the C57BL/6J (RRID:IMSR_JAX:000664) genetic background. Wild‐type (WT) mice were on a C57BL/6J background, and their genotype was *Card9* ^+/+^. C57BL/6J mice employed in this study were sourced from the Institute of Model Animals, Nanjing University. Mice were allocated randomly into four groups (*n* = 5 per group). Male mice aged eight weeks were employed in this present study, as detailed in the respective figure descriptions, and were distributed at random into experimental groups. Prior to treatment, body mass did not differ significantly among the groups. Following the completion of the experiment, euthanasia was performed via carbon dioxide inhalation. All animal experiments were complied with relevant ethical regulations.

To detect the potential effect of *Card9* in MASLD, WT male mice and *Card9* ^−^ male mice aged 8 weeks were assigned to two groups through randomization. WT CD and *Card9* ^−^ CD groups of mice received a chow diet (CD), whereas those in the WT HFD and *Card9* ^−^ HFD‐ groups were provided a high‐fat diet (HFD) for a duration of 12 weeks.

To further confirm the role of mycobiota in MASLD, the fecal microbiota transplants (FMT) experiment was performed. For donor mice, WT HFD groups mice and *Card9* ^−^ HFD groups mice received HFD for a duration of 12 weeks. In weeks 6–12, feces (20 mg) obtained from HFD mice were pooled and suspended in a volume of 1 mL of phosphate‐buffered saline (PBS), vortexed for 3 min, and then spun at 200 × *g* over 3 min under 4°C. The resulting separated fluid was then obtained for subsequent use. Self‐FMT group mice received HFD for a duration of 12 weeks. Mice received antibiotic treatment for 1 week at week 5. Mice received an anti‐bacterial antibiotic mixture in their drinking water (vancomycin at 0.5 mg mL^−1^, ampicillin at 1 mg mL^−1^, neomycin at 1 mg mL^−1^, and metronidazole at 1 mg mL^−1^) along with oral administration of the antifungal agent Amphotericin B (23 µg/mouse) by gavage every 3 days. In weeks 6–12, mouse feces were collected as described above. For recipient mice, C57BL/6J mice were maintained on an HFD over a 12‐week duration. At the 5th week, for seven days, mice received an anti‐bacterial antibiotic mixture in their drinking water (vancomycin at 0.5 mg mL^−1^, ampicillin at 1 mg mL^−1^, neomycin at 1 mg mL^−1^, and metronidazole at 1 mg mL^−1^) along with oral administration of the antifungal agent Amphotericin B (23 µg/mouse) by gavage every 3 days. In the 6th week, mice treated with an antibiotic mixture and Amphotericin B received fecal microbiota transplants (100 µL/mice) from donor mice every other day for 7 weeks. Both donor and recipient mice used for FMT experiments were 5 mice per group. To prevent alterations in the microbial makeup, fresh transplant material was readied within a timeframe of 10 min prior to oral gavage. Mice from the same experimental group were housed together, while different experimental groups were housed in separate cages throughout the study. Donor and recipient mice were maintained in separate cages.

To explore whether the gut mycobiota influenced the progress of *Card9* deficiency‐associated MASLD, 8‐week‐old WT male mice and *Card9* ^−^ male mice were each separated at random into two groups. HFD groups of mice were given HFD, meanwhile, treated with DMSO by gavage every 3 days for 12 weeks. HFD+Amphotericin B groups of mice were offered HFD, meanwhile, administered antifungal Amphotericin B (23 µg/mice) by gavage every 3 days for 12 weeks.

To examine the effect of *S. cerevisiae* on the progression of MASLD in vivo, WT male mice and *Card9* ^−^ male mice at 8 weeks of age were randomized into 3 groups. Mice in the WT CD and *Card9* ^−^ CD groups were maintained on a chow diet (CD), meanwhile gavaged with PBS for 12 weeks, mice in the WT HFD and *Card9* ^−^ HFD groups received high‐fat diet (HFD), meanwhile gavaged with PBS for 12 weeks, WT HFD+*S. cerevisiae* and *Card9* ^−^ HFD+*S. cerevisiae* groups of mice were fed with HFD meanwhile received 1 × 10^8^ CFU of *S. cerevisiae* delivered in 200 µL PBS by oral gavage every 3 days for 12 weeks.

To clarify whether 5‐HIAA participates in the *Card9*‐deficiency‐mediated MASLD progression in vivo, WT male mice and *Card9* ^−^ male mice aged 8 weeks were each divided at random into three groups. Mice in WT CD and *Card9* ^−^ CD groups were fed with a chow diet (CD), meanwhile gavaged with PBS for 12 weeks, mice in WT HFD and *Card9* ^−^ HFD groups were treated with a high‐fat diet (HFD) and simultaneously administered PBS by gavage for 12 weeks. WT HFD+5‐HIAA and *Card9* ^−^ HFD+5‐HIAA groups were fed with HFD, meanwhile treated with 5‐HIAA by gavage (0.5 mg/mouse, every 3 days) for 12 weeks.

To test the potential role of *S. cerevisiae* and 5‐HIAA in MASH in the MCD model, WT male mice aged 8 weeks were randomly assigned to four groups. WT CD group mice were fed with chow diet (CD) meanwhile gavaged with PBS for 8 weeks, WT MCD group mice were maintained on a methionine–choline‐deficient (MCD) diet meanwhile gavaged with PBS lasting 8 weeks, mice in the WT MCD + *S. cerevisiae* group were maintained on the MCD diet and, at the same time, received 1 × 10^8^ CFU of *S. cerevisiae* suspended in a volume of 200 µL of PBS and administered via oral gavage every 3 days for 8 weeks, WT HFD+5‐HIAA group mice received an MCD diet meanwhile treated with 5‐HIAA by gavage (0.5 mg/mouse, every 3 days) for 8 weeks.

To further explore the protective effect of *S. cerevisiae* against MASLD via TLR1 in vivo, WT male mice and *Tlr1* ^−^ male mice with an age of 8 weeks were each divided at random into three groups. Mice in WT CD and *Tlr1* ^−^ CD groups were fed with a chow diet (CD), meanwhile gavaged with PBS for 12 weeks, mice in WT HFD and *Tlr1* ^−^ HFD groups were treated with a high‐fat diet (HFD) and simultaneously administered PBS by gavage for 12 weeks, mice in WT HFD+*S. cerevisiae* and *Tlr1* ^−^ HFD+*S. cerevisiae* groups were fed with HFD, meanwhile, received 1 × 10^8^ CFU of *S. cerevisiae* delivered in 200 µL PBS by oral gavage every 3 days for 12 weeks.

To explore whether the gut bacteria influenced the progress of *Card9* deficiency‐associated MASLD, 8‐week‐old WT male mice and *Card9* ^−^ male mice were each separated at random into two groups. HFD groups of mice were given HFD and normal drinking water for 12 weeks. HFD+ABX groups of mice were offered HFD, meanwhile administered an anti‐bacterial antibiotic mixture in their drinking water (vancomycin at 0.5 mg mL^−1^, ampicillin at 1 mg mL^−1^, neomycin at 1 mg mL^−1^, and metronidazole at 1 mg mL^−1^) for 12 weeks.

To further confirm the specific role of *S. cerevisiae* in *Card9* deficiency‐associated MASLD, WT male mice and *Card9* ^−^ male mice with an age of 8 weeks were each divided at random into three groups. Mice in WT HFD and *Card9* ^−^ HFD groups were treated with a high‐fat diet (HFD) for 12 weeks. Mice in WT HFD+AmB and *Card9* ^−^ HFD+AmB groups were also fed an HFD for 12 weeks, and during weeks 1–6, they were administered Amphotericin B (AmB, 23 µg/mouse) by gavage every 3 days. Amphotericin B treatment was discontinued from weeks 7 to 12. Mice in WT HFD+AmB+*S. cerevisiae* and *Card9* ^−^ HFD+AmB+*S. cerevisiae* groups were also with a HFD for 12 weeks, and during weeks 1–6, they were administered AmB by gavage every 3 days. From weeks 7 to 12, AmB treatment was discontinued, and the mice were orally gavaged with *S. cerevisiae* (1 × 10^8^ CFU) in 200 µL PBS every 3 days.

To further investigate whether heat‐killed *S. cerevisiae* can alleviate MASLD, heat‐killed *S. cerevisiae* was prepared by heat treatment of the cell suspension at 95°C for 45 min. WT male mice aged 8 weeks were divided at random into four groups. Mice in WT CD were fed with a chow diet (CD), meanwhile gavaged with PBS for 12 weeks, mice in WT HFD were treated with a high‐fat diet (HFD) and simultaneously administered PBS by gavage for 12 weeks, mice in WT HFD+*S. cerevisiae* groups were fed with HFD, meanwhile received 1 × 10^8^ CFU of *S. cerevisiae* delivered in 200 µL PBS by oral gavage every 3 days for 12 weeks, mice in WT HFD+HK‐*S. cerevisiae* groups were fed with HFD; meanwhile, they received 1 × 10^8^ CFU of *HK‐S. cerevisiae* was delivered in 200 µL PBS by oral gavage every 3 days for 12 weeks.

To further investigate the regulation of 5‐HIAA production by *S. cerevisiae in vivo*, WT male mice at the age of 8 weeks were divided at random into four groups. Mice in WT CD were fed with a chow diet (CD) for 12 weeks, mice in WT HFD were treated with a high‐fat diet (HFD) for 12 weeks, mice in WT HFD+*S. cerevisiae* groups were fed with HFD, meanwhile, received 1 × 10^8^ CFU of *S. cerevisiae* delivered in 200 µL PBS by oral gavage every 3 days for 12 weeks, mice in the WT HFD+*S. cerevisiae*+clorgyline group were fed an HFD and administered *S. cerevisiae* (1 × 10^8^ CFU) in 200 µL PBS by oral gavage every 3 days, plus clorgyline (30 mg/kg) by intraperitoneal injection every 3 days, for 12 weeks.

### 4.3 Cell Lines and Cell Culture

AML‐12, a normal mouse hepatocyte cell line (ATCC: CRL‐2254; RRID:CVCL_0140), human colon epithelial cells Coca‐2 cells (ATCC: HTB‐37; RRID:CVCL_0025), and HEK293T, a human embryonic kidney cell line (ATCC: CRL‐3216; RRID:CVCL_0063) were obtained from Jiangsu KeyGEN Biotech Co., Ltd. (Jiangsu, China). AML‐12 cells were maintained in DMEM/F‐12 (Gibco) containing 10% fetal bovine serum (FBS), insulin (5 mg mL^−1^), transferrin (5 µg mL^−1^), selenium (5 ng mL^−1^), dexamethasone (40 ng mL^−1^), and 1% penicillin–streptomycin (Thermo Fisher Scientific). Caco‐2 and HEK293T cells were maintained in DMEM medium (Gibco) containing 10% fetal bovine serum (FBS) and 1% penicillin–streptomycin. Cells were cultured in a 5% CO_2_ incubator at 37°C.

### 4.4 16S rDNA Gene Sequencing and Analysis

#### 4.4.1 Isolation and Sequencing of DNA

Genomic DNA derived from mouse fecal samples was isolated employing the E.Z.N.A. Stool DNA Kit as per the manufacturer's guidelines. This kit, which is specifically formulated to isolate DNA from very small quantities of material, has been demonstrated to efficiently isolate DNA from diverse bacterial taxa. Ultrapure water free of nucleic acids served as the negative control. The extracted DNA was resuspended in a volume of 50 µL of elution buffer and kept at −80°C prior to PCR analysis.

Following DNA extraction, the V3–V4 segments of 16S rRNA in prokaryotes, including bacteria and archaea, were PCR‐amplified using slightly modified primers 341F (5'‐CCTACGGGNGGCWGCAG‐3') and 805R (5'‐GACTACHVGGGTATCTAATCC‐3'). Sample‐specific barcodes and universal sequencing primers were added to the 5' ends of the primers. Amplification was conducted in a 25 µL mixture consisting of 25 ng template DNA, 12.5 µL PCR Premix, 2.5 µL of each primer, with PCR‐grade water added to reach 25 µL. The amplification program for the prokaryotic 16S rRNA fragments included an initial denaturation at 98°C for 30 s, followed by 32 cycles of 98°C for 10 s (denaturation), 54°C for 30 s (annealing), and 72°C for 45 s (extension), with a final extension at 72°C lasting 10 min. PCR products were verified on an agarose gel at 2% concentration. Ultrapure water was employed instead of a sample during DNA extraction as a blank control to rule out incorrect positive signals. The amplified PCR products were purified via AMPure XT beads (Beckman Coulter Genomics, Danvers, MA, USA) and their concentrations measured using Qubit (Invitrogen, USA). Amplicon libraries were prepared for subsequent sequencing, with library size and concentration evaluated using the Agilent 2100 Bioanalyzer (Agilent, USA) and the Illumina Library Quantification Kit (Kapa Biosciences, Woburn, MA, USA), respectively. PhiX Control v3 (Illumina) was added to the amplicon library at a final proportion of 30%. Sequencing of the libraries was performed on 300 PE MiSeq runs; one library followed both protocols with standard Illumina primers, obviating additional index reads.

#### 4.4.2 Analysis of Sequencing Data

Paired‐end reads were merged into continuous sequences and subjected to accuracy filtering using PANDAseq (v2.9) to filter out reads shorter than 220 nucleotides or longer than 500 nucleotides, sequences exhibiting a mean quality score under 20, or those containing more than three ambiguous bases. Filtered sequences were assigned to OTUs at 97% similarity using UPARSE (v7.0) within QIIME (v1.8), and UCHIME was used to identify and discard chimeric sequences. Using a 70% confidence threshold, OTUs were classified taxonomically with the RDP classifier against the SILVA 16S rRNA database (Release 128). Alpha diversity, including the Shannon index and OTU counts per sample, was calculated with MOTHUR (v1.30.1). Visual representations, including bar plots and heat maps, were produced using the R package “vegan” (v3.3.1). Beta diversity, calculated from Bray–Curtis distances, was analyzed via principal component analysis (PCA) using QIIME (v1.8).

### 4.5 ITS rDNA Gene Sequencing and Analysis

#### 4.5.1 Isolation and Sequencing of DNA

Total microorganism‐derived DNA from fecal samples was isolated using the Fecal Genome DNA Extraction Kit (DP712‐02, TIANGEN, China) as per the provided guidelines. DNA concentration was determined with a Qubit fluorometer (Invitrogen, USA). PCR was executed on the total DNA with the broad‐range primers ITS1FI2 and ITS2 (ITS1FI2:5′‐GTGARTCATCGAATCTTTG‐3′; ITS2:5′‐TCCTCCGCTTATTGATATGC‐3′). PCR procedure was carried out with a preliminary denaturation at 95°C lasting 30 s, subsequently undergoing 32 cycles of 95°C over 10 s (denaturation), 52°C over 30 s (annealing), and 72°C over 45 s (extension), with a concluding elongation at 72°C over 10 min. The resulting PCR products were purified with AMPure XT Beads (Beckman Coulter Genomics, Danvers, MA, USA) and quantified using a Qubit fluorometer (Invitrogen, USA). After assessment with an Agilent 2100 Bioanalyzer (Agilent, USA) and Illumina library quantification kits (Kapa Biosciences, Woburn, MA, USA), qualified products were pooled and sequenced on an Illumina NovaSeq 6000 platform (PE250) at LC‐Bio Technology Co., Ltd., Hangzhou, China.

#### 4.5.2 ITS Sequencing Data Processing and Analysis

Raw reads were first demultiplexed, and sequencing primers were removed using cutadapt (v1.9). The resulting paired‐end reads were subsequently merged with FLASH (v1.2.8). Reads with average quality scores below 20, lengths under 100 bp, or containing more than 5% ambiguous nucleotides (“N”) were filtered out using the sliding‐window approach in fqtrim (v0.94) to obtain high‐quality clean sequences. Chimeric sequences were then detected and eliminated using Vsearch (v2.3.4). Finally, DADA2 was employed to denoise the dataset and produce amplicon sequence variants (ASVs). Species annotation was conducted using the QIIME2 feature‐classifier plugin, with RDP and UNITE serving as the reference databases. Alpha and beta diversity analyses were performed using QIIME2, and fungal taxonomy was assessed based on relative abundance. The Wilcoxon test was used to identify differentially abundant classes, with *p* < 0.05 indicating significance.

### 4.6 Fungal Burden Assay

The overall fungal burden and individual fungal species were assessed by qPCR according to established protocols [89, 90]. Fecal specimens from experimental mice were subjected to incubation at 37°C for 30 min following suspension in a lytic solution composed of 50 mM Tris (pH 7.5), 1 m EDTA, 0.2% β‐mercaptoethanol, and 1000 U/mL lyticase. Fungal genomic DNA was subsequently harvested using the QIAamp DNA Stool Mini Kit in accordance with the manufacturer's guidelines. For quantification of fungal ITS2 rDNA, fecal DNA (100 ng) served as the template for qPCR analysis. Species‐specific fungal detection was conducted on genomic DNA with the corresponding primers shown in Table S3. The overall fungal load was measured using the ΔCt approach and standardized to both fecal sample weight and input DNA quantity. The relative proportion of individual fungal species was also determined using the ΔCt method, and the percentage of individual fungi was expressed as the fraction of the contribution of individual species to overall fungal burden.

### 4.7 Untargeted Metabolomics Analysis

Samples remained on ice until fully thawed, and metabolites were obtained in prechilled methanol (80%). Specifically, a volume of 100 µL of portal vein blood was blended with a volume of 400 µL of methanol, while feces or liver (50 mg) were suspended and homogenized in 0.5 mL of methanol. The homogenates were kept at −20°C for over 30 min, then spun down at 20 000 × g for 15 min. The resulting liquid was collected into fresh tubes and dried under vacuum. Each sample was reconstituted in a volume of 100 µL of methanol (80%) and preserved at ultra‐low temperature (−80°C) for LC–MS‐based analysis. A total of 10 µL from each extract was pooled to prepare Quality Control (QC) samples.

LC–MS data acquisition was performed according to the instrument protocol. UPLC analysis was performed on an UltiMate 3000 system (Thermo Fisher Scientific, Bremen, Germany) with a 100 mm × 2.1 mm, 1.8 µm ACQUITY UPLC T3 column (Waters, Milford, USA) at 40°C. The eluent included solvent A (5 mm solution of ammonium acetate and 5 mm solution of acetic acid in water) and solvent B (acetonitrile), flowed at 0.3 mL/min. Ramped elution was programmed as: 2% B from 0 to 0.8 min; linearly increased to 70% B over 0.8–2.8 min; then to 90% B over 2.8–5.6 min; further ramped to 100% B from 5.6–6.4 min and maintained until 8.0 min; subsequently decreased back to 2% B from 8.0–8.1 min and held at 2% B until 10 min.

Metabolite profiling was conducted on a Q‐Exactive high‐resolution tandem mass spectrometer (Thermo Scientific) utilizing dual ionization modes (positive and negative). Full MS data were collected from *m/z* 70–1050 with a resolving power of 70,000, with an AGC target of 3 × 10^6^ and a 100 ms upper limit for injection time. MS/MS spectra were obtained using a Top 3 data‐dependent acquisition strategy, with the resolution set to 17 500, an AGC target of 1 × 10^5^, and an ion injection time of 80 ms. Pooled QC samples, generated from all study samples, were analyzed following each set of ten injections to assess the stability of the instrument.

Raw LC–MS data were converted to mzXML format and processed using the XCMS, CAMERA, and metaX toolboxes in R. Data processing steps included peak detection, grouping, retention time alignment, secondary grouping, and annotation of isotopes and adducts. Each ion was characterized by its retention time (RT) and *m/z* value. Peak intensities were recorded to construct a 3D matrix comprising peak indices (RT–*m/z* pairs), sample identifiers, and corresponding ion intensity values.

Metabolite annotation was performed by comparing the precise molecular masses (*m/z*) of the samples with records from the KEGG and HMDB databases. Metabolites were identified when the observed masses deviated from database values by less than 10 ppm. Molecular formulas were further determined and verified by comparing isotopic distribution patterns. Additionally, metabolite identification was confirmed using an in‐house MS/MS spectral library.

Data analyses were carried out in R (version 4.0.0). Raw protein signal values were normalized using a median‐based approach. Tree‐based clustering was carried out using the pheatmap, while principal component analysis (PCA) was carried out through metaX. PLS‐DA was conducted via the ropls package, with VIP scores calculated for every feature. Correlations were assessed by applying Pearson's correlation through the cor package. Metabolites showing *p* < 0.05 (Student's t‐test), FC > 1.2, and VIP > 1 were deemed significantly different. Enrichment analysis of KEGG pathways was carried out via the hypergeometric test. Gene set enrichment analysis (GSEA) was conducted with GSEA v4.1.0 and MSigDB to evaluate whether specific KEGG pathway‐associated genes were significantly differentially expressed across groups. Significance criteria were set as |NES| > 1, nominal *p* < 0.05, and FDR *q* < 0.25. Network maps were constructed based on pathways associated with the identified metabolites.

### 4.8 Metabolic Cage Procedures and Analytical Approaches

Each mouse was housed separately in the Promethion High‐Definition Multiplexed Respirometry System (Sable Systems International, North Las Vegas, NV, USA). The housing cages were situated within environmental chambers with controlled temperature and lighting (DB034‐LT Laboratory Incubator, Darwin Chambers Company, St. Louis, MO, USA). Animals were kept under a 12‐h light/dark photoperiod at 22°C, provided with pine chip bedding, food, and acidified water provided without restriction, except where noted. Indirect calorimetry was applied to determine energy expenditure, calculated according to the Weir equation [91]. Data from respirometry were captured at 5‐min intervals, with each cage monitored for 30 s and baseline measurements taken every 30 s across four cages. Oxygen consumption (VO_2_) and carbon dioxide production (VCO_2_) were measured every second, and the respiratory exchange ratio (RER) was determined as the CO_2_/O_2_ ratio. Nutrient and water consumption, as well as body mass, were measured through weighing. Locomotor activity was assessed by XY position displacement using beam disruptions (PedMeters and AllMeters). Data were assessed using CalR with the dual‐groups template. Whole‐body energy expenditure, RER, food intake, and activity data were assessed by two‐factor repeated‐measures ANOVA. Associations across variables were evaluated by Spearman's rank correlation [92].

### 4.9 Ultrasound Attenuation Analysis (USAT)

USAT examinations were performed by a specialist blinded to the patients’ clinical diagnoses. Expert USAT assessments were conducted with the Resona 7 system (Mindray, probe SC6‐1U, Shenzhen, China). All subjects underwent a fasting period exceeding 8 h. Participants were positioned supine during the scan, with their right arm placed over their head. The specialist positioned the probe within the right hepatic lobe, ensuring the upper boundary of the sampling frame was 1–2 cm beneath the liver capsule. USAT readings were recorded when the sampling frame changed to yellow, with results expressed in dB/cm/MHz.

### 4.10 MRI Protocol

The MRI protocol involved iterative decomposition of water and fat with echo asymmetry and least squares estimation with a 3.0‐T MRI scanner (Architect; GE HealthCare) for liver fat quantification. The scan parameters were as follows: repetition time, 6.4 msec; first echo time, 0.9 msec; six echoes with delta echo time, 0.8 msec; field of view, 440 × 330 mm; section thickness, 3 mm; flip angle, 3°; matrix, 160 × 160 × 50; excitation number, 0.5; and scan time, 18 s. For each examination, three 2–3‐cm^2^ circular regions of interest (ROIs) were placed in the peripheral area of the left, right anterior, and right posterior lobes, avoiding major blood vessels, bile ducts, and artifacts. Mean PDFF values (percentage) were calculated for each ROI.

### 4.11 Isolation of Primary Hepatocytes

Primary hepatocytes from mice were obtained through enzymatic perfusion with collagenase of the inferior vena cava (IVC) [93]. Before administering collagen‐digesting enzyme, the liver was pre‐flushed for 3–4 min through the IVC following ligation of the hepatic portal vein. Collagenase perfusion was carried out for 2 min until the liver surface began to show light cracking, indicating sufficient degradation. The liver was subsequently removed and positioned in pre‐chilled buffer. Cells were passed through a 100 µm cell strainer and spun at 60 × g for 6 min under 4°C conditions. Cells were washed a single time using buffer without collagenase, resuspended, and spun using identical conditions. Cells were then resuspended in Percoll and spun at 100 × g for 10 min. The hepatocyte sediment was harvested, rinsed one time with buffer, and maintained in culture medium enriched with penicillin, streptomycin, and 10% FBS. Following incubation for approximately 16 h, the medium was substituted with extracellular vesicle‐free Dulbecco's Modified Eagle Medium (DMEM) enriched with antibiotics.

### 4.12 Seahorse Assay

Mitochondrial stress was evaluated through the Seahorse XF Cell Mito Stress Test Kit (Agilent Technologies) in line with the instructions. In brief, cells were plated in XF96 microplates at 4 × 10^4^ cells per well and kept at incubation conditions overnight. For the assay, the existing medium was swapped for 180 µL of XF basal medium adjusted to pH 7.4, comprising glucose at a concentration of 10 m, glutamine at a concentration of 2 m, and pyruvate at a concentration of 1 m, and the plates were kept at 37°C without CO_2_ for 60 min. Oligomycin at a concentration of 1.5 µ, FCCP at a concentration of 1 µ, and Rotenone/Antimycin A at a concentration of 0.5 µ were loaded into the sensor cartridge injection ports. OCR was measured sequentially after the addition of each compound, and values were adjusted according to protein content and analyzed using Wave software.

### 4.13 *Saccharomyces cerevisiae* Strain and Culture

*Saccharomyces cerevisiae* was purchased from BeNa Culture Collection (Henan, China): 336054. *Saccharomyces cerevisiae* was cultured at 30°C in yeast malt extract medium and shaked cultivation at 200 rpm/min to expand overnight. For oral administration in mice, *Saccharomyces cerevisiae* was cultured in yeast malt extract medium, collected, and reconstituted in sterile PBS to an end concentration of 1 × 10^8^ CFU mL^−1^ under aseptic conditions. Each mouse was administered 200 µL by gavage. Colony‐forming units (CFU/mL) were quantified using a dilution plating method on Yeast Malt Extract Agar.

### 4.14 Fluorescent Labeling and Tracing of *S. cerevisiae*

*S. cerevisiae* (100 million CFU) was exposed to 0.5 m Cy5‐NHS ester lasting 6 h, followed by washing, and then delivered to HFD‐fed mice via oral gavage. Five hours after administration, colons were excised, fixed in 4% paraformaldehyde, and submerged for approximately 14 h at 4°C in 30% (w/v) sucrose for cryoprotection. The tissues were then embedded in OCT compound (Leica, 14020108926), cut into 10 µm‐thick cryosections, and affixed with DAPI Fluoromount‐G (SouthernBiotech, 0100–20). Images were captured on an Andor Dragonfly high‐speed spinning disk confocal system and subsequently processed using ImageJ software (RRID:SCR_003070).

### 4.15 Co‐Culture of *S. cerevisiae* and Caco‐2

Caco‐2 cells were plated in 12‐well plates (10^6^ cells/well). Cells were serum starved for 8 h and maintained in medium containing 10% FBS, 1% penicillin–streptomycin, and L‐Tryptophan (2 mg/mL) (HY‐N0623, MCE, USA). Then Caco‐2 cells were exposed to *S. cerevisiae* (MOI = 0.6) for 3 h/6 h, followed by other experimental assays. *S. cerevisiae* was cultured in yeast malt extract medium, collected, and resuspended in sterile PBS.

### 4.16 Co‐Culture of Primary Hepatocytes With Caco‐2 and *S. cerevisiae*

Primary hepatocytes obtained from mice were grown in 12‐well culture vessels at 1 × 10^5^ cells/well. After a one‐night incubation, cells were exposed to free fatty acids (1 mm/L) (P5585‐10G, O1008‐5G, Sigma, Germany) for 48 h. After 48 h, fresh culture medium with L‐tryptophan (2 mg mL^−1^) was added to replace the old medium. And cells were exposed to Caco‐2 cells (10^5^ cells/well) along with *S. cerevisiae* (MOI = 0.6) for 12 h with/without clorgyline hydrochloride addition.

### 4.17 Intraperitoneal Glucose Tolerance Test (ipGTT) and Intraperitoneal Insulin Tolerance Test (ipITT)

Glucose and insulin testing were performed as described [94]. For the intraperitoneal glucose tolerance test (ipGTT), mice were deprived of food for 16 h, and then intraperitoneal administration of glucose (2 g/kg) was administered using a 31‐gauge insulin syringe. Measurements of glycemia were taken at 0, 30, 60, and 120 min through tail vein sampling using an Accu‐Check glucometer (Bayer, Leverkusen, Germany). The intraperitoneal insulin tolerance test (ipITT) was conducted following a 6‐h fast, with glucose measurements taken at 0, 30, 60, and 120 min subsequent to insulin being administered intraperitoneally (1.5 IU/kg). Additionally, fasting blood glucose levels were assessed from samples collected after a 16‐h fast.

### 4.18 Measurements of Serum and Liver Biochemical Markers

Circulating concentrations of aspartate aminotransferase (AST), alanine aminotransferase (ALT), total triglycerides (TG), total cholesterol (TC), and non‐esterified fatty acids (NEFA) were determined using assay kits obtained from Nanjing Jiancheng Institute of Biotechnology (#C010‐2‐1, #C009‐2‐1, #A110‐1‐1, #A111‐1‐1, #A042‐2‐1, Jiangsu, China) in line with the guidelines. Hepatic TG content was determined using kits from the same supplier (#A110‐1‐1). For this, homogenization of liver tissue (50 mg) was performed with a mixture of methanol and chloroform (2:1, v/v) and collected at room temperature overnight, with the resulting organic phase used for TG measurement. Intracellular TG levels were assessed using the same Nanjing Jiancheng kit (#A110‐1‐1), while intracellular free fatty acids were quantified with a kit from Beijing Solarbio Science & Technology Co., Ltd. (#BC0595, Beijing, China).

### 4.19 Bodipy Staining for Confocal Fluorescence Microscopy

Fixation of cells was performed using 4% paraformaldehyde (PFA) for 10 min, then rinsed three times with PBS, allowing 5 min per wash. The fixed cells were then incubated with BODIPY 493/503 (5 µg/mL in PBS, shielded from light; Invitrogen, D3922) for 30 min. After staining, samples were rinsed three PBS washes and subsequently incubated with DAPI for 10 min for counterstaining.

### 4.20 Bodipy Staining for Flow Cytometry

Rinse the cells briefly with 3 mL of PBS to eliminate residual media and serum. Then, cells were kept at 37°C in dark conditions with BODIPY staining solution for 15 min. A brief wash with a volume of 3 mL of PBS was performed to eliminate the dye solution from the cells. Trypsinize cells to produce a single cell suspension. Add 5 mL of PBS and transfer the cell suspension to a 15 mL conical tube. Pellet cells at 300 × g, 5 min, 4°C. Aspirate supernatant, wash the cell pellet with a quick rinse using 3 mL PBS, and pellet cells at 300 × g, 5 min, 4°C. Carefully aspirate the supernatant and resuspend cells in 300 µL 1 ×flow cytometry buffer. Pass cell suspension through a 35 µm filter into a FACS tube. Perform flow cytometry.

### 4.21 Histological Analysis

Liver tissue was examined histologically using H&E staining, using 4 µm sections embedded in paraffin. Two independent investigators, blinded to experimental groups, evaluated the histological features. The extent of steatosis and inflammation was assessed through previously established criteria [95], and the MASLD/MASH functional score was determined by summing the scores of steatosis, inflammation, and hepatocyte ballooning. Steatosis was additionally verified through Oil Red O staining on cryosections, and the areas positive for Oil Red O were quantified using Image J (RRID:SCR_003070).

### 4.22 Quantification of 5‐HIAA and 5‐HT

The concentrations of 5‐HIAA and 5‐HT were assessed using mouse‐specific ELISA kits (Elabscience, E‐EL‐0075c and E‐EL‐0033c, Wuhan, China) following the manufacturer's protocol. These assays are based on a competitive ELISA format, and the resulting colorimetric signals were recorded at 450 nm with the aid of a microplate reader.

### 4.23 Celluar TG and Oil Red O Staining

Cellular triglyceride (TG) levels were assessed using an assay kit from Nanjing Jiancheng Institute of Biotechnology (#A110‐1‐1, Jiangsu, China) in line with the manufacturer's guidelines. Cells underwent three washes with PBS and were then preserved in formaldehyde (3.7%) for 20 min. Oil Red O (0.5% in isopropanol) was made up with water at a 3:2 ratio and applied to the fixed cells and kept at ambient temperature for 2 h. Subsequent to washing with water, lipid bodies within the cells were visualized under a light microscope and photographed.

### 4.24 Molecular Docking

For the docking of AhR (receptor molecule) and 5‐HIAA (Ligand Molecule). We use the docking tool on Autodock Vina software (RRID:SCR_011958). The structure of 5‐HIAA was obtained via the PubChem (pubchem.ncbi.nlm.nih.gov). Protein structure of AhR downloaded from the PDB (https://www.rcsb.org/).

### 4.25 Cellular Thermal Shift Assay (CETSA)

AML12 cells were cultured in a 10 cm dish and isolated with RIPA to collect total proteins, then incubated with 5‐HIAA (20 µ) or DMSO at 37°C for 4 h. All samples were heated at different temperatures (from 45°C to 70°C) for 3 min and quickly cooled with ice. The soluble components were separated from lysates by centrifugation, and the protein expression of AhR was ultimately detected by western blotting.

### 4.26 Immunofluorescence Staining

To localize AhR in hepatocytes, cells were first preserved with paraformaldehyde (4%) at ambient temperature for 15 min, after which they were rinsed three times with PBS. A 10‐min treatment with 0.1% Triton X‐100 was applied to permeabilize the cells. To avoid nonspecific interactions, cells were exposed to 5% BSA dissolved in PBS at ambient temperature for 1 h. Subsequently, cells were exposed to primary antibodies against AhR (Proteintech, 67785‐1‐Ig; RRID:AB_2918549) under 4°C for 16–18 h. After thorough PBS rinses, secondary fluorescent antibodies (Proteintech, SA00013‐1; RRID:AB_2810983) were applied in the dark for 1 h. Nuclei were labeled with DAPI at 1 µg mL^−1^ over 5 min. Finally, cover glasses were set with ProLong Gold Antifade Mountant, and laser scanning confocal microscopy (FV3000, Olympus) was employed for imaging.

### 4.27 In Vitro Transfection and Luciferase Experiments

Vectors (RRID:Addgene_48743) containing the Renilla luciferase reporter fused to a segment of the CPT1A/ACOX1 3′‐UTR with predicted AhR binding sites were co‐transfected into HEK‐293T cells along with AhR mimic (Hesheng Technology, China). For transient transfections, cells were plated in 24‐well culture vessels (10^5^ cells/well). After 24 h, cells were transfected with Lipofectamine 2000 (Invitrogen, USA) transfection reagent. At 24 h after plating, 500 ng of firefly luciferase reporter plasmid, 200 ng of pGL3‐basic expression vector, and 200 ng of AHR mimic were transfected into cells with Lipofectamine 2000 (Invitrogen, USA) according to the manufacturer's instructions. A pGL3‐basic plasmid served as the control for transfection. Following 4 h, the medium was replaced with normal DMEM medium. Luciferase activity was assessed 48 h post‐transfection using a Promega (USA) kit. Each experimental condition was assessed in triplicate wells, and the entire experiment was independently repeated three times.

### 4.28 Transfection of siRNA

Cells were introduced with siRNA (Hesheng Technology, China) using Lipofectamine 2000 (Invitrogen, USA) in Opti‐MEM medium without antibiotics or serum, following the protocol. A non‐targeting siRNA control (si‐NC) served as the mock control. After 4 h, the transfection medium was substituted with standard DMEM, and cells were maintained until used in subsequent experiments. SiRNA sequences designed to target *Ahr* were as listed below: Sense, 5’‐GCAGAUGCCUUGGUCUUCUAUGCUU‐3’; Antisense, 3′‐AAGCAUAGAAGACCAAGGCAUCUGC‐5′. SiRNA sequences designed to target *Cpt1a* were as listed below: Sense, 5’‐ CCAUGGAUCUGCUGUAUAUTT‐3’; Antisense, 3’‐ AUAUACAGCAGAUCCAUGGTT‐5’. SiRNA sequences designed to target *Acox1* were as listed below: Sense, 5’‐CCUGAAGAAGCUGUACUAAUTT‐3’; Antisense, 3’‐AUUAGUACAGCUUAUUCAGGTT‐5’. SiRNA sequences designed to target *TLR1* were as listed below: Sense, 5'‐GCAGAUUGUCAAGGUGAAATT‐3'; Antisense, 3'‐AAGCACUUCACCUUGACAAAUCUGC‐5'. SiRNA sequences designed to target *TLR9* were as listed below: Sense, 5'‐GCAGAUUGACAGAAGAACUUGCUUU‐3'; Antisense, 3'‐AAAGCAAGUUCUUCUGUCAAUCUGC‐5'. SiRNA sequences designed to target *Dectin‐1* were as listed below: Sense, 5'‐GCAGAUGACCUCAAGUACUUCAUCUU‐3'; Antisense, 3'‐AAGAUGAAGUACUUGAGGUCATCUGC‐5'. SiRNA sequences designed to target *NOD2* were as listed below: Sense, 5'‐GCAGAUGCUGGAAGAAGAUCUUCUUU‐3'; Antisense, 3'‐AAAGAAGAUCUUCUUCCAGCAUCUGC‐5'.

### 4.29 Chromatin Immunoprecipitation (ChIP) Assays

ChIP assays were performed using a Chromatin Immunoprecipitation (ChIP) Kit (BersinBio) according to the manufacturer's protocol. Subsequently, to measure the DNA fragments that coimmunoprecipitated with anti‐p65 antibody, qPCR analysis was performed with special primers flanking the potential *Mao‐a* gene binding sites. The sequences of primers are shown in Table S3.

### 4.30 Cell Counting Kit‐8 Assay

Hepatocyte expansion was assessed by a CCK‐8 kit (Dojindo, Shanghai, China). In short, 1000 cells in a volume of 100 µL of medium encompassing 10% FBS were plated per well in a 96‐well plate with eight replicates. An operational solution was freshly made by integrating CCK‐8 reagent (10 µL) with DMEM (90 µL), and 100 µL of this operational solution was dispensed to every well, after which the samples were maintained for 1.5 h. Optical density at 450 nm (OD450) was subsequently measured with a microplate reader.

### 4.31 Real‐Time Quantitative PCR

The entire RNA fraction was obtained from mouse liver or cells propagated in vitro by the FastpPure Cell/Tissue Total RNA Isolation Kit (Vazyme, #RC112‐01) in line with guidelines. The amount and purity of RNA were determined with a Nanodrop 2000, and cDNA was generated from 1000 ng of RNA by reverse transcription using HiScript RT SuperMix (Vazyme, #R323‐01) with gDNA Eraser. Quantitative real‐time PCR was executed using SYBR Green Master Mix (Applied Biosystems, #A25742) on an Applied Biosystems instrument using Viia 7 software. Primer sequences are provided in Table S3.

### 4.32 Protein Isolation and Western Blotting

Tissue and cell extracts were homogenized in ice‐cold RIPA buffer with protease and phosphatase inhibitors. Protein concentration was determined by the BCA protein assay [23], and proteins were distinguished on 8%–10% SDS‐polyacrylamide gels and transferred onto a PVDF membrane. Following a 5% nonfat milk blocking protocol, the PVDF membrane underwent incubation for ∼16 h at 4°C conditions with the specific primary antibody prepared in TBST containing 5% BSA, using gentle orbital shaking. Three washes of the membrane with TBST were performed at room temperature, each lasting 8 min. Next, the membrane was exposed to the corresponding secondary antibody, also prepared in TBST including 5% BSA, for 2 h at ambient temperature under light stirring, followed by three additional TBST washes of 8 min each at room temperature.

Primary antibodies were purchased from Proteintech: CPT1A (15184‐1‐AP; RRID:AB_2084676), ACAT1 (16215‐1‐AP; RRID:AB_2220210), ACOX1 (68017‐1‐Ig; AB_2918763), GAPDH (60004‐1‐Ig; RRID:AB_2107436). MCAD antibody purchased from Abcam (ab92461; RRID:AB_10563530). H3 (17168‐1‐AP; RRID:AB_2716755), Tubulin (11224‐1‐AP;RRID:AB_2210206).

### 4.33 Statistical Analysis

The data were presented as mean ± SD and analyzed using GraphPad Prism 8.0 software (GraphPad Software, CA, USA). Two‐tailed, unpaired Student's *t*‐tests were retained only for comparisons between two groups, while one‐way or two‐way ANOVA followed by Tukey's multiple comparisons test was applied when more than two groups were analyzed. Correlation significance was determined by using linear regression. Differences were considered significant at *p* < 0.05. ^*^ *p* ≤ 0.05; ^**^ *p* ≤ 0.01; ^***^ *p* ≤ 0.001.

## Author Contributions

S.Q. methodology, formal analysis, data acquisition, project administration, study design, and writing – review & editing. S.F. statistical analysis and methodology. J.X. clinical data collection, software, and statistical analysis. Z.X. data acquisition, validation, software, and funding acquisition. C.P. statistical analysis and methodology. J.Q. methodology and software. Z.B. methodology and funding acquisition. S.Z. data analysis and software. S.S. formal analysis, software, and management. G.X. project management, study design, and manuscript editing. Y.Z. manuscript editing and funding acquisition. T.W. idea formulation, management, study concept, and experimental design.

## Ethics Statement

This study is authorized by the Ethics Committee of the Medical School of Nanjing University and the Hospital of Integrated Traditional Chinese and Western Medicine, Nanjing University of Chinese Medicine.

## Conflicts of Interest

The authors declare no conflicts of interest.

## Supporting information




**Supporting File 1**: advs75268‐sup‐0001‐SuppMat.pdf.

**Supporting File 2**: advs75268‐sup‐0002‐Data.zip.

## Data Availability

The raw ITS and 16S sequencing data generated for the human and murine experiments have been deposited in the NCBI Sequence Read Archive under the accession numbers PRJNA1422771, PRJNA1420243, PRJNA1420185, and PRJNA1427889. The liver RNA‐seq data are deposited in the Gene Expression Omnibus (GSE318635). Non‐targeted metabolomic data are available at MetaboLights (MTBLS13895).
