# Supplementary figures and images for: Gut Mycobiota‐Associated Tryptophan Catabolites Protect Against Metabolic Dysfunction‐Associated Steatotic Liver Disease
Source: Adv Sci (Weinh). 2026 Apr 29;13(39):e14830. doi: 10.1002/advs.202514830 (PMC13334940; doi:10.1002/advs.202514830)

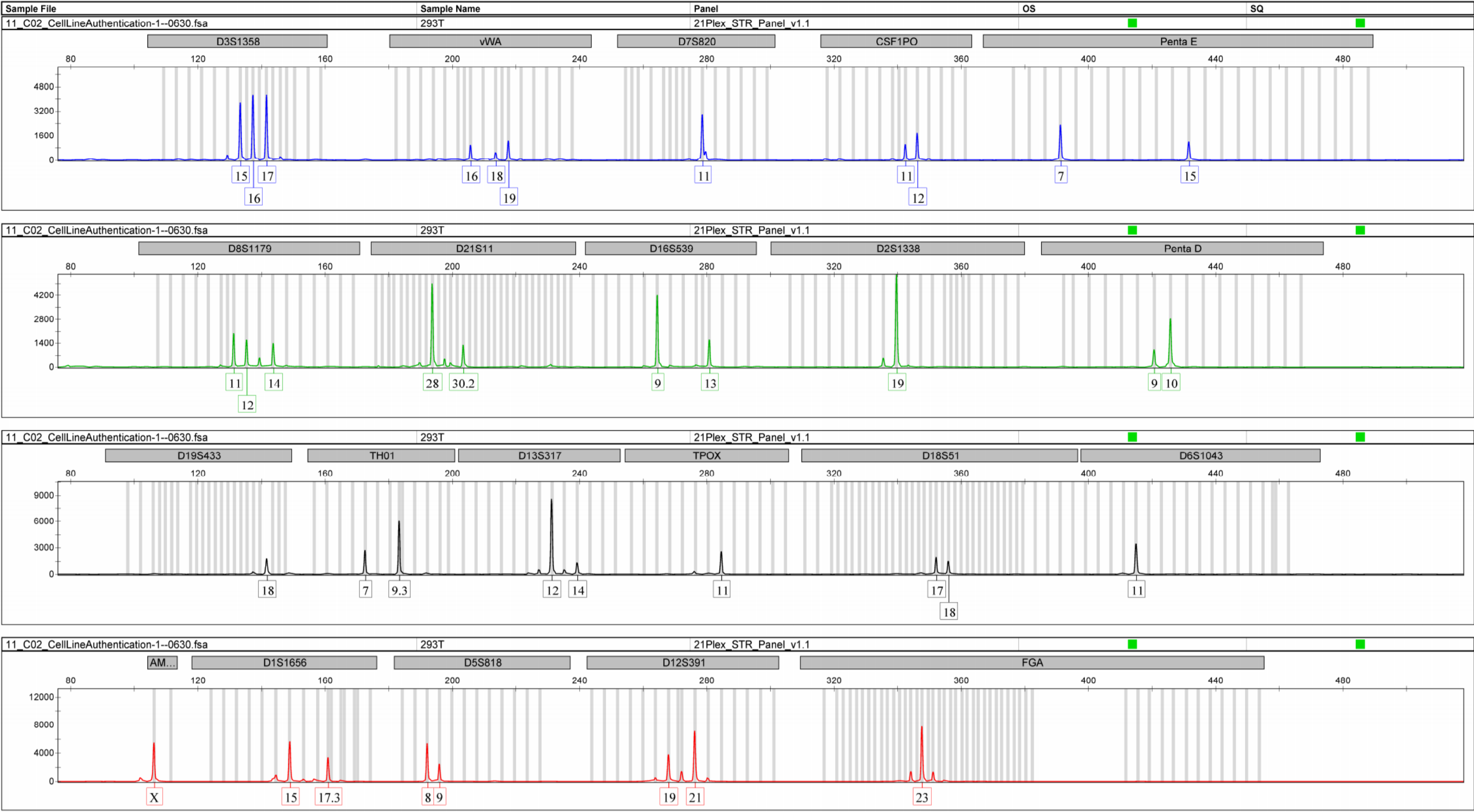

Supplement: Supplementary file 2 — Supporting File 2: advs75268‐sup‐0002‐Data.zip. [file ADVS-13-e14830-s001.zip › STR profiling of HEK293T cell line.pdf]
