# Supplementary material for: Gut Mycobiota‐Associated Tryptophan Catabolites Protect Against Metabolic Dysfunction‐Associated Steatotic Liver Disease
Source: Adv Sci (Weinh). 2026 Apr 29;13(39):e14830. doi: 10.1002/advs.202514830 (PMC13334940; doi:10.1002/advs.202514830)

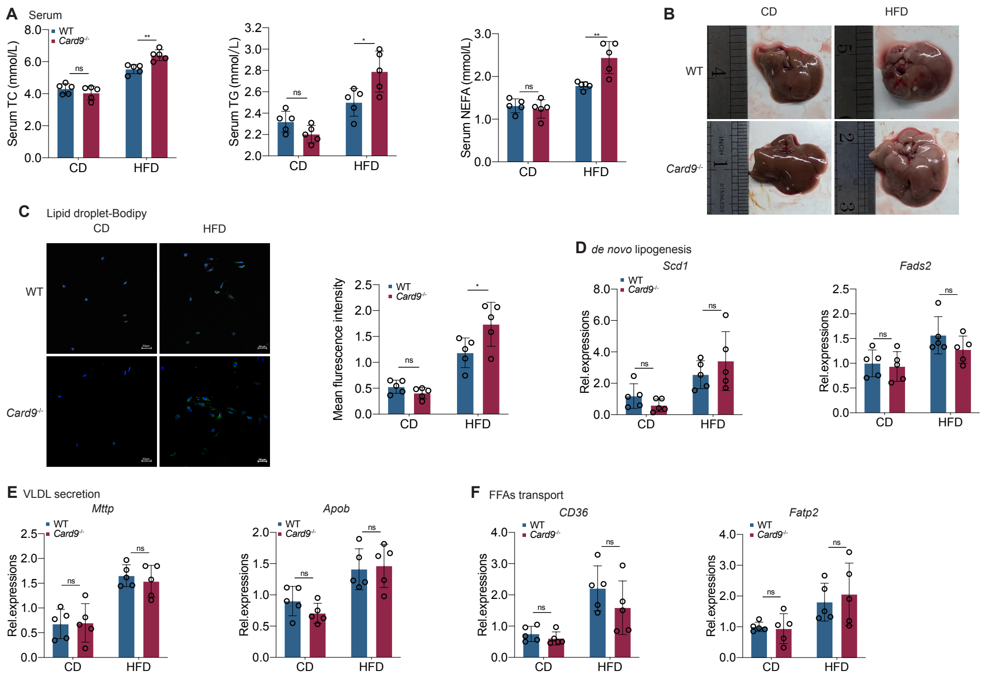

Figure S1. ***Card9**$^{-/-}$* **mice have severe MASLD upon HFD treatment than WT mice.**

A. Levels of TC, TG and NEFA in serum. **B.** Representative macroscopic appearance of livers. **C.** Lipid droplets in primary hepatocytes of mice discovered by BODIPY 493/503 (green) fluorescent dye staining. Scale bar=50μm. Quantification of mean fluorescence intensity. **D.** Q-PCR analysis of relative mRNA expression levels of genes involved in *de novo* lipogenesis (*Scd1, Fads2*) in mice livers. **E.** Q-PCR analysis of relative mRNA expression levels of genes involved in VLDL secretion (*Mttp, Apob*) in mice livers. **F.** Q-PCR analysis of relative mRNA expression levels of genes involved in FFAs transport (*CD36, Fatp2*) in mice livers. Each dot represents data from individual biological replicate. Biological replicate numbers in each group are as follows: (Figure S1A-F) n=5. Data were shown as mean ± SEM. *p < 0.05, **p < 0.01, ***p < 0.001 as determined by two-way ANOVA followed by Tukey's multiple comparison (**A** and **C-F**).

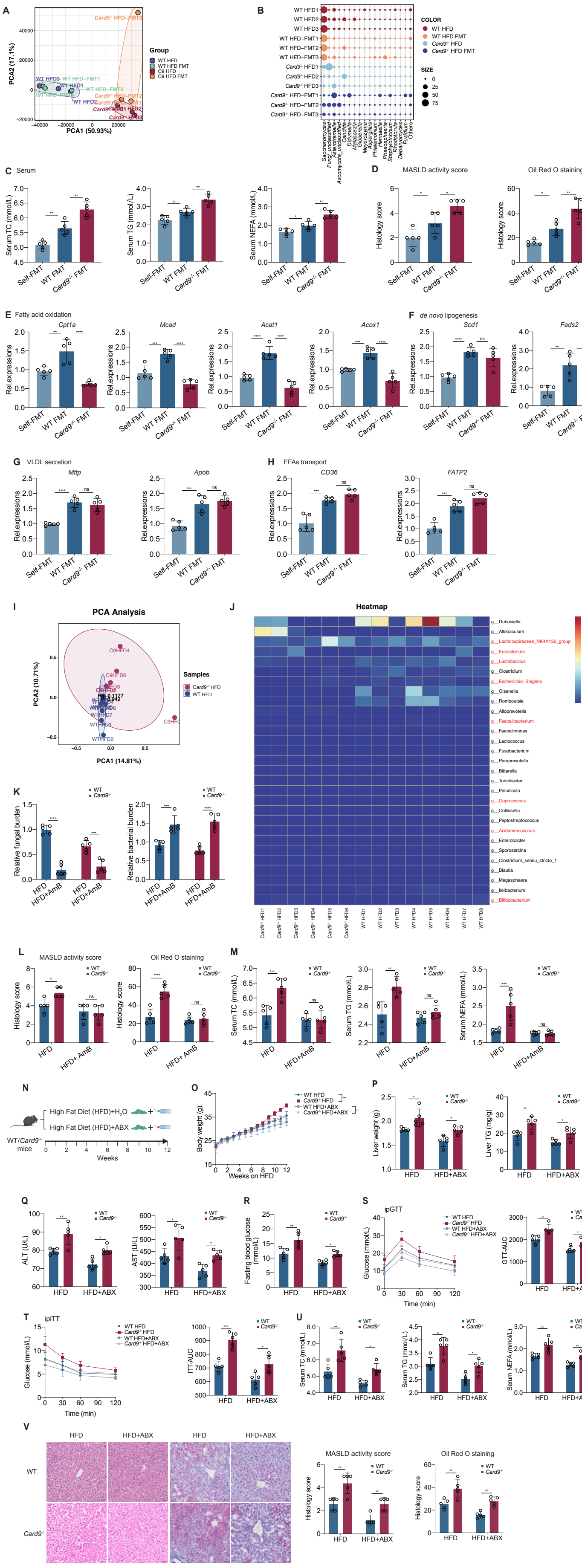
A
PCA2 (17.1%)
PCA1 (50.93%)
Group
WT HFD
WT HFD FMT
C9 HFD
C9 HFD FMT
B
COLOR
WT HFD
WT HFD FMT
Card9⁻/⁻ HFD
Card9⁻/⁻ HFD FMT
SIZE
C Serum
Serum TC (mmol/L)
Serum TG (mmol/L)
Serum NEFA (mmol/L)
Self-FMT
WT FMT
Card9⁻/⁻ FMT
D MASLD activity score
Oil Red O staining
Histology score
E Fatty acid oxidation
Cpt1a
Mcad
Acat1
Acox1
Rel.expressions
F de novo lipogenesis
Scd1
Fads2
G VLDL secretion
Mttp
Apob
H FFAs transport
CD36
FATP2
I
PCA Analysis
PCA2 (10.71%)
PCA1 (14.81%)
Samples
Card9⁻/⁻ HFD
WT HFD
J
Heatmap
K
Relative fungal burden
Relative bacterial burden
HFD
HFD+AmB
L
MASLD activity score
Oil Red O staining
M
N
High Fat Diet (HFD)+H₂O
High Fat Diet (HFD)+ABX
WT/Card9⁻/⁻ mice
Weeks
O
Body weight (g)
Weeks on HFD
P
Liver weight (g)
Liver TG (mg/g)
HFD
HFD+ABX
Q
ALT (U/L)
AST (U/L)
R
Fasting blood glucose (mmol/L)
S
ipGTT
Glucose (mmol/L)
Time (min)
GTT-AUC
T
ipITT
ITT-AUC
U
Serum TC (mmol/L)
Serum TG (mmol/L)
Serum NEFA (mmol/L)
V
WT
Card9⁻/⁻
MASLD activity score
Oil Red O staining

13

Figure S2. Impact of CARD9-mediated gut mycobiota disorder in HFD-induced MASLD.

A. Weighted UniFrac PCA analysis based on fungal ITS2 gene sequence in the feces. **B.** The relative abundances of different fungal genera based on fungal ITS2 gene sequence. **C.** Levels of TC, TG and NEFA in serum. **D.** MASLD activity score quantification and Oil Red O positive area quantification. **E.** Q-PCR analysis of relative mRNA expression levels of genes involved in fatty acid oxidation (*Cpt1a*, *Mcad, Acat1*, *Acox1*) in mice livers. **F.** Q-PCR analysis of relative mRNA expression levels of genes involved in *de novo* lipogenesis (*Scd1, Fads2*) in mice livers. **G.** Q-PCR analysis of relative mRNA expression levels of genes involved in VLDL secretion (*Mttp, Apob*) in mice livers. **H.** Q-PCR analysis of relative mRNA expression levels of genes involved in *FFAs transport* (*CD36, Fatp2*) in mice livers. **I.** Weighted UniFrac PCA analysis based on bacteria 16s rDNA gene sequence in the feces. **J.** The relative abundances of different bacteria genera based on bacteria 16s rDNA gene sequence. **K.** The fungi burden in feces of HFD-fed WT mice and HFD-fed $Card9^{-/-}$ mice with/without gavaging Amphotericin B. **L.** MASLD activity score quantification and Oil Red O positive area quantification. **M.** Levels of TC, TG and NEFA in serum. **N.** Experimental schematic of WT and $Card9^{-/-}$ mice fed with High Fat Diet (HFD) for 12 weeks with/without ABX treatment. **O.** Body weight changes during the 12-week experimental period. **P.** Liver weight of mice in different groups (left) and content of Triglyceride (TG) in mice liver in different groups (right). **Q.** Levels of ALT and AST in serum. **R.** The glucose concentration at fasting levels. **S.** Levels of serum glucose for intraperitoneal glucose tolerance tests (ipGTT) (left) and area under the curve (AUC) (right). **T.** Levels of serum glucose for intraperitoneal insulin tolerance tests (ipITT) (left) and area under the curve (AUC) (right). **U.** Levels of TC, TG and NEFA in serum. **V.** Representative liver histology by H&E staining (up) and Oil Red O staining (down). Scale bars=50μm. MASLD activity score quantification and Oil Red O positive area quantification. Each dot represents data from individual biological replicate. Biological replicate numbers in each group are as follows: (Figure S2A-B) n=3, (Figure S2C-H, K-M, O-V) n=5, (Figure S2I-J) n=6-8. Data were shown as mean ± SEM. *p < 0.05, **p < 0.01, ***p < 0.001 as determined by one-way ANOVA followed by Tukey's multiple comparison (**C-H**) or two-way ANOVA followed by Tukey's multiple comparison (**K-M**, **O-V**).

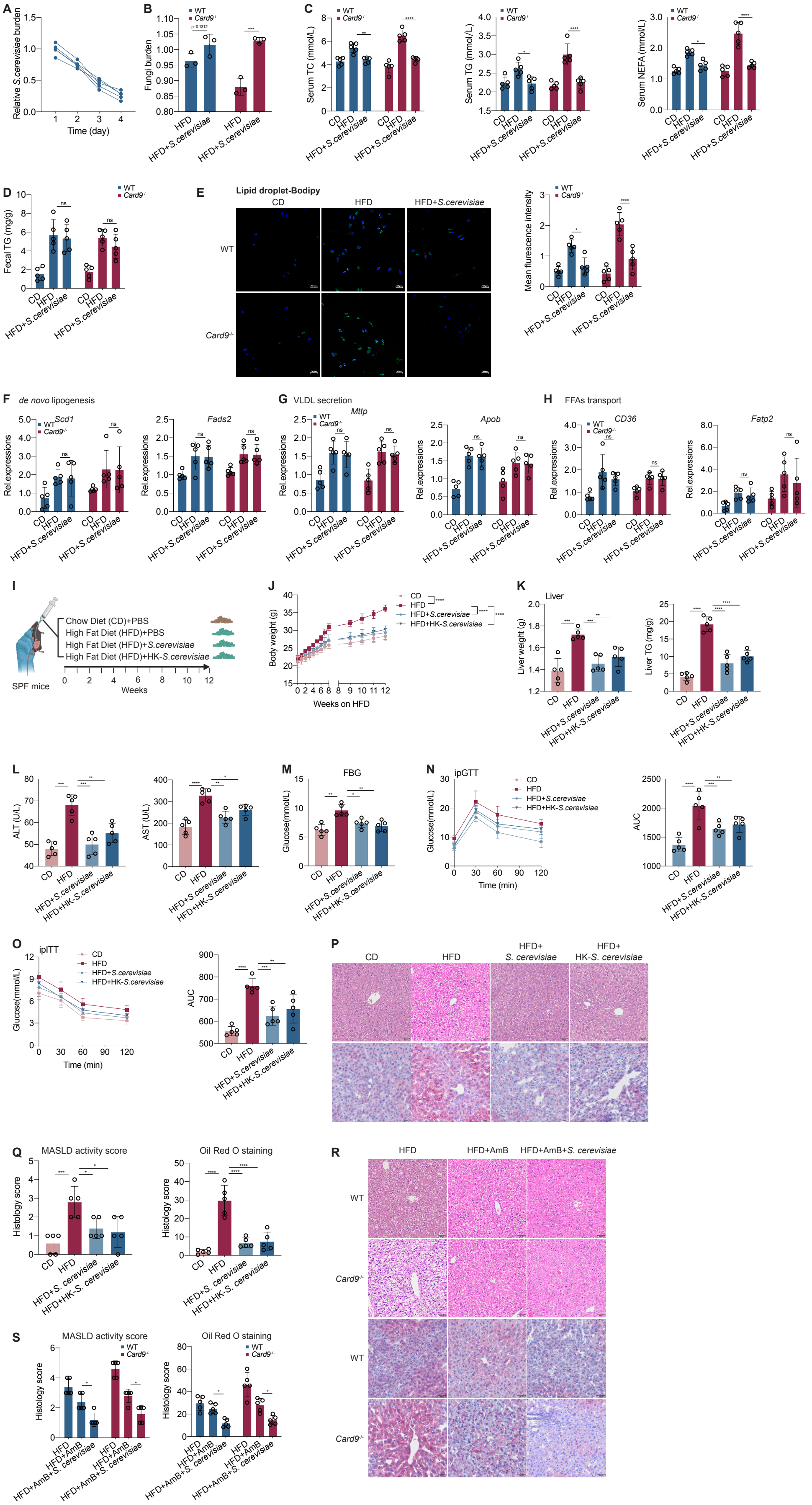
A
Relative S.cerevisiae burden
Time (day)
B
Fungi burden
WT
Card9-/-
p=0.1312
HFD
HFD+S.cerevisiae
C
Serum TC (mmol/L)
Serum TG (mmol/L)
Serum NEFA (mmol/L)
CD
D
Fecal TG (mg/g)
ns
E
Lipid droplet-Bodipy
HFD+S.cerevisiae
Mean fluorescence intensity
F
de novo lipogenesis
Scd1
Fads2
Rel.expressions
G
VLDL secretion
Mttp
Apob
H
FFAs transport
CD36
Fatp2
I
Chow Diet (CD)+PBS
High Fat Diet (HFD)+PBS
High Fat Diet (HFD)+S.cerevisiae
High Fat Diet (HFD)+HK-S.cerevisiae
SPF mice
Weeks
J
Body weight (g)
Weeks on HFD
HFD+HK-S.cerevisiae
K
Liver
Liver weight (g)
Liver TG (mg/g)
L
ALT (U/L)
AST (U/L)
M
FBG
Glucose(mmol/L)
N
ipGTT
Time (min)
AUC
O
ipITT
P
HFD+
S. cerevisiae
HK-S. cerevisiae
Q
MASLD activity score
Oil Red O staining
Histology score
HFD+S. cerevisiae
HFD+HK-S. cerevisiae
R
HFD+AmB
HFD+AmB+S. cerevisiae
S

Figure S3. Beneficial role of *S. cerevisiae* in *Card9* deficiency associated MASLD.

A. Relative *S. cerevisiae* burden in HFD-fed WT mice feces detecting by Q-PCR. **B.** The fungi burden in feces of HFD-fed WT mice and HFD-fed *Card9*$^{-/-}$ mice with/without gavaging *S. cerevisiae* detecting by Q-PCR**. C.** Levels of TC, TG and NEFA in serum. **D.** Triglyceride (TG) content in mouse feces. **E.** Lipid droplets in primary hepatocytes of mice. Scale bar=50 μm. Quantification of mean fluorescence intensity. **F.** Q-PCR analysis of relative mRNA expression levels of genes involved in *de novo* lipogenesis (*Scd1, Fads2*) in mice livers. **G.** Q-PCR analysis of relative mRNA expression levels of genes involved in VLDL secretion (*Mttp, Apob*) in mice livers. **H.** Q-PCR analysis of relative mRNA expression levels of genes involved in FFAs transport (*CD36, Fatp2*) in mice livers. **I.** Experimental schematic of WT mice fed with Chow Diet (CD)/High Fat Diet (HFD) for 12 weeks. *S. cerevisiae* or HK-*S. cerevisiae* was gavaged to HFD mice every 3 days. **J.** Body weight changes during the 12-week experimental period. **K.** Liver weight of mice in different groups (left) and content of Triglyceride (TG) in mice liver in different groups (right). **L.** Levels of ALT and AST in serum. **M.** The glucose concentration at fasting levels. **N.** Levels of serum glucose for intraperitoneal glucose tolerance tests (ipGTT) (left) and area under the curve (AUC) (right). **O.** Levels of serum glucose for intraperitoneal insulin tolerance test (ipITT) (left) and area under the curve (AUC) (right). **P.** Representative liver histology by H&E staining (up) and Oil Red O staining (down). Scale bars=50μm. **Q.** MASLD activity score quantification and Oil Red O positive area quantification. **R.** Representative liver histology by H&E staining (up) and Oil Red O staining (down). Scale bars=50μm. **S.** MASLD activity score quantification and Oil Red O positive area quantification. Each dot represents data from individual biological replicate. Biological replicate numbers in each group are as follows: (Figure S3A) n=4, (Figure S3B) n=3, (Figure S3C-H, J-S) n=5. Data were shown as mean ± SEM. *p < 0.05, **p < 0.01, ***p < 0.001 as determined by two-way ANOVA followed by Tukey's multiple comparison (**B-H** and **S**) or one-way ANOVA followed by Tukey's multiple comparison (**J-O** and **Q**).

A

Fecal

Metabolites	WT HFD	*Card9*$^{-/-}$ HFD
Isoleucyl-Threonine	6.8041358	18.55544457
Ursodeoxycholic acid	2038.06485	5131.155979
Oleic acid	92.2703875	220.2699222
**5-Hydroxyindoleacetic acid**	7.90376886	2.096534721
L-Lactic acid	176.608405	420.0988157
L-Phenylalanine	314.137484	738.433705
Cinnamic acid	20.2798693	47.04363405
Uric acid	37.6390258	85.74952881
Xanthine	298.876458	628.7309129
13-OxoODE	10.057437	4.761777401

B

Portal vein blood

Metabolites	WT HFD	*Card9*$^{-/-}$ HFD
Homovanillic acid	131.162404	60.24784017
4-Pyridoxic acid	23.5065447	10.74789563
2-Methylhippuric acid	59.6366194	22.22566795
Isovalerylglycine	153.900194	46.88216716
Oxidized glutathione	423.783574	120.2016572
Glycine	15.5838748	4.169515948
17-Hydroxymethylethisterone	8.88871924	2.378205487
**5-Hydroxyindoleacetic acid**	11.2412854	3.001752103
Valerylglycine	18.7176256	4.674810913
N-lactoyl-Leucine	63.748588	15.42520754

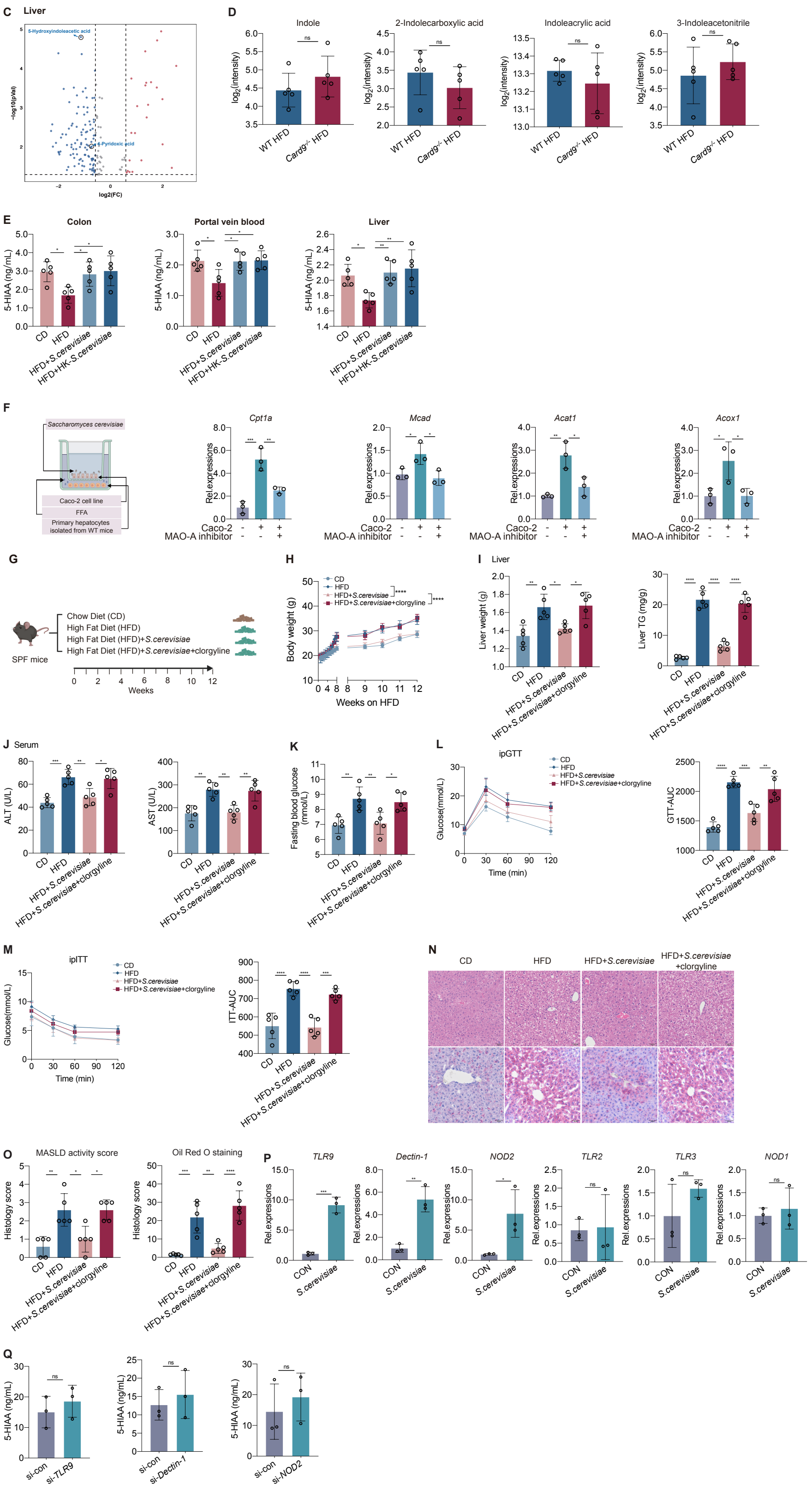

61

Figure S4. ***S. cerevisiae*** **induces tryptophan catabolites 5-HIAA production in intestine.**
A-C. The different metabolites in fecal, portal vein blood and liver separately between HFD-fed WT mice and HFD-fed *Card9*$^{-/-}$ mice. **D.** Relative levels of indoles, 2-indolecarboxylic acid, indoleacrylic acid, and 3-indoleacetonitrile in the liver of HFD-fed mice and HFD-fed *Card9*$^{-/-}$ mice based on Untargeted metabolomic analysis. **E.** The content of 5-HIAA in colon, portal vein blood and liver. **F.** Schematics for co-culture of *S. cerevisiae*, Caco-2 cells and primary hepatocytes which were treated with free fatty acid for 48h. Q-PCR analysis of relative mRNA expression levels of fatty acid oxidation genes (*Cpt1a, Mcad*, *Acat1*, *Acox1*). **G.** Experimental schematic of WT mice fed with Chow Diet (CD)/High Fat Diet (HFD) for 12 weeks. *S. cerevisiae* was gavaged to HFD mice every 3 days. Clorgyline was administered intraperitoneally every 3 days. **H.** Body weight changes during the 12-week experimental period. **I.** Liver weight of mice in different groups (left) and content of Triglyceride (TG) in mice liver in different groups (right). **J.** Levels of ALT and AST in serum. **K.** The glucose concentration at fasting levels. **L.** Levels of serum glucose for intraperitoneal glucose tolerance tests (ipGTT) (left) and area under the curve (AUC) (right). **M.** Levels of serum glucose for intraperitoneal insulin tolerance test (ipITT) (left) and area under the curve (AUC) (right). **N.** Representative liver histology by H&E staining (up) and Oil Red O staining (down). Scale bars=50μm. **O.** MASLD activity score quantification and Oil Red O positive area quantification. **P.** Q-PCR analysis of relative mRNA expression levels of fungal recognition receptors genes in Caco-2 cells (*TLR9*, *Dectin-1*, *NOD2*, *TLR2, TLR3* and *NOD1*). **Q.** The content of 5-HIAA with different fungal receptor knockdown. Each dot represents data from individual biological replicate. Biological replicate numbers in each group are as follows: (Figure S4A-E, G-O) n=5, (Figure S4F, P-Q) n=3. Data were shown as mean ± SEM. *$p < 0.05$, **$p < 0.01$, ***$p < 0.001$ as determined by two-tailed, unpaired Student's t-test (**D** and **P-Q**), one-way ANOVA followed by Tukey's multiple comparison (**E-F, H-M** and **O**).

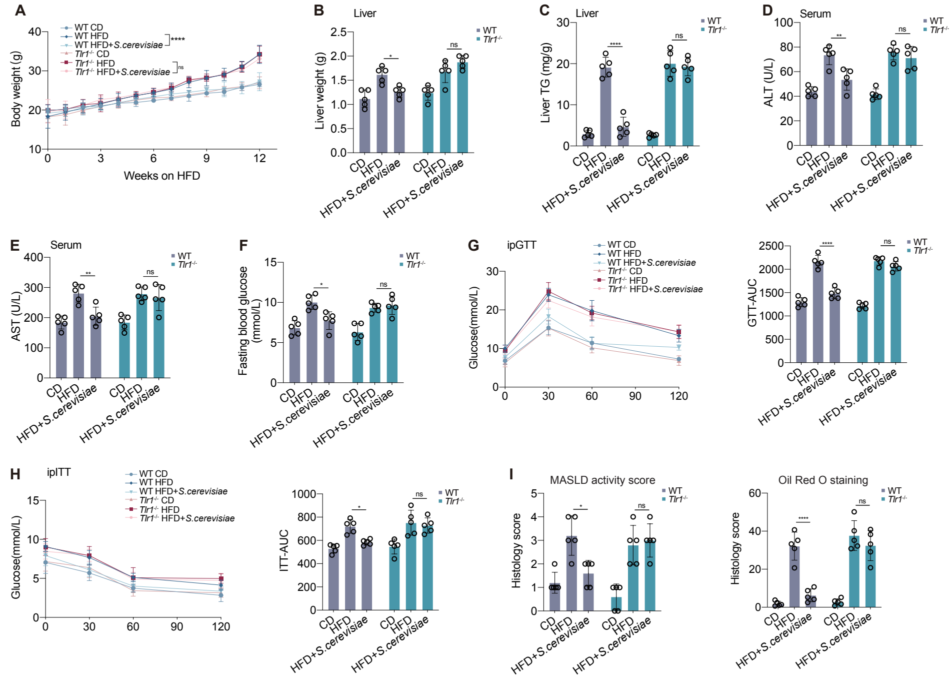

Figure S5. ***S. cerevisiae*** **ameliorates** ***Card9$^{-/-}$*****-associated MASLD via TLR1.**

A. Body weight changes during the 12-week experimental period. **B.** Liver weight of mice in different groups. **C.** The content of Triglyceride (TG) in mice liver in different groups. **D.** Levels of ALT in serum. **E.** Levels of AST in serum. **F.** The glucose concentration at fasting levels. **G.** Levels of serum glucose for intraperitoneal glucose tolerance tests (ipGTT) (left) and area under the curve (AUC) (right). **H.** Levels of serum glucose for intraperitoneal insulin tolerance test (ipITT) (left) and area under the curve (AUC) (right). **I.** MASLD activity score quantification and Oil Red O positive area quantification. Each dot represents data from individual biological replicate. Biological replicate numbers in each group are as follows: (Figure 5A-I) n=5. Data were shown as mean ± SEM. *p < 0.05, **p < 0.01, ***p < 0.001 as determined by two-way ANOVA followed by Tukey's multiple comparison (**A-I**).

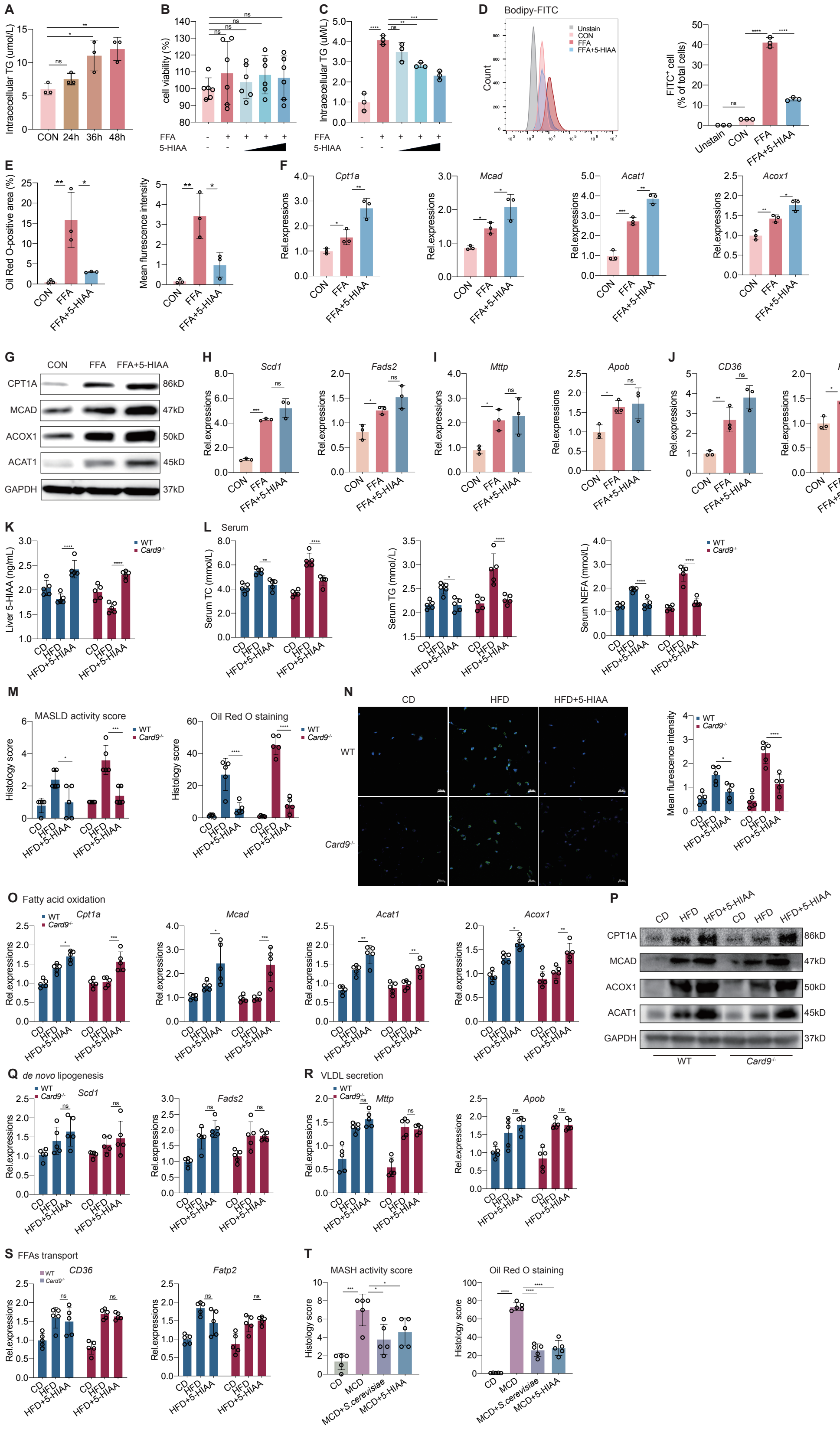

Figure S6. Beneficial role of 5-HIAA in *Card9* deficiency associated MASLD.

A. The content of TG in hepatocytes with FFAs treatment in different time (24h/36h/48h). **B.** The cell viability with 5-HIAA treatment in different concentration (62.5, 125 and 250 μm/L). **C.** The content of TG in hepatocytes with

5-HIAA treatment in different concentration (62.5, 125 and 250 μm/L). **D.** Lipid droplets measured by flow cytometry (left), FITC-positive cells of total cells were quantified (right). **E.** Oil Red O positive area quantification (left) and mean fluorescence intensity quantification (right). **F.** Q-PCR analysis of relative mRNA expression levels of genes involved in fatty acid oxidation (*Cpt1a*, *Mcad*, *Acat1, Acox1*) in hepatocytes treated by 5-HIAA. **G.** Western blot analysis of relative protein involved in fatty acid oxidation (CPT1A, MCAD, ACOX1, ACAT1) in hepatocytes treated by 5-HIAA. GAPDH was used as the internal standard. **H.** Q-PCR analysis of relative mRNA expression levels of genes involved in *de novo* lipogenesis (*Scd1*, *Fads2*) in hepatocytes treated by 5-HIAA. **I.** Q-PCR analysis of relative mRNA expression levels of genes involved in VLDL secretion (*Mttp*, *Apob*) in hepatocytes treated by 5-HIAA. **J.** Q-PCR analysis of relative mRNA expression levels of genes involved in FFAs transport (*CD36, Fatp2*) in hepatocytes treated by 5-HIAA. **K.** The content of 5-HIAA in liver. **L.** Levels of TC, TG and NEFA in serum. **M.** MASLD activity score quantification and Oil Red O positive area quantification. **N.** Lipid droplets in primary hepatocytes of mice discovered by BODIPY 493/503 (green) fluorescent dye staining. Scale bar=50μm. Quantification of mean fluorescence intensity. **O.** Q-PCR analysis of relative mRNA expression levels of genes involved in fatty acid oxidation (*Cpt1a*, *Mcad*, *Acat1*, *Acox1*) in mice livers. **P.** Western blot analysis of relative protein involved in fatty acid oxidation (CPT1A, MCAD, ACOX1, ACAT1) in mice livers. GAPDH was used as the internal standard. **Q.** Q-PCR analysis of relative mRNA expression levels of genes involved in *de novo* lipogenesis (*Scd1*, *Fads2*) in mice livers. **R.** Q-PCR analysis of relative mRNA expression levels of genes involved in VLDL secretion (*Mttp*, *Apob*) in mice livers. **S.** Q-PCR analysis of relative mRNA expression levels of genes involved in FFAs transport (*CD36, Fatp2*) in mice livers. **T.** MASH activity score quantification and Oil Red O positive area quantification. Each dot represents data from individual biological replicate. Biological replicate numbers in each group are as follows: (Figure S6A, C-J) n=3, (Figure S6B) n=6, (Figure S6K-T) n=5. Data were shown as mean ± SEM. *$p < 0.05$, **$p < 0.01$, ***$p < 0.001$ as determined by one-way ANOVA followed by Tukey's multiple comparison (**A-F, H-J** and **T**) or by two-way ANOVA followed by Tukey's multiple comparison (**K-O** and **Q-S**).

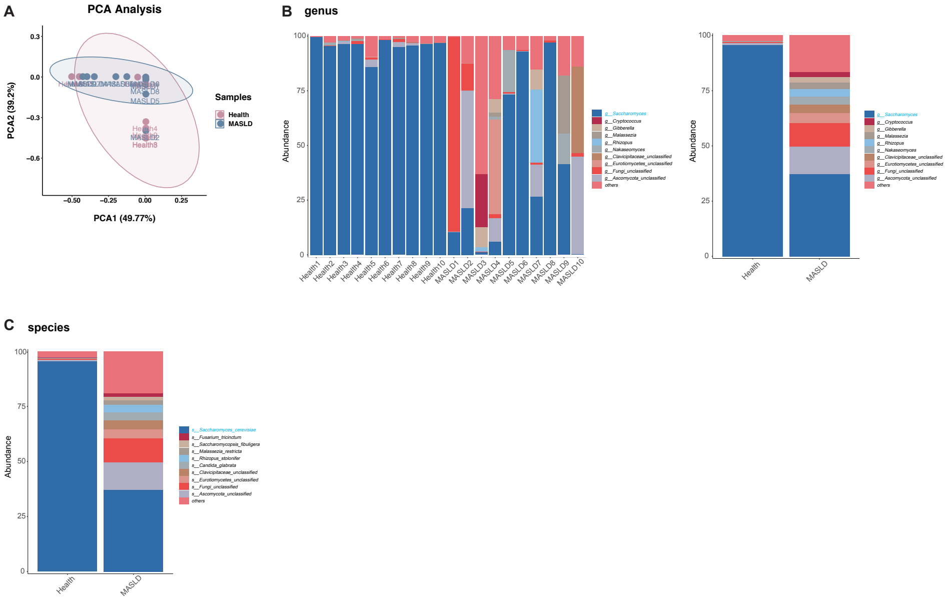

Figure S7. Fungal ITS sequencing for feces from health individuals and MASLD patients. **A.** Weighted UniFrac PCA analysis. **B.** Barplot analysis of the genus level of the gut microbial community in the feces of healthy individuals and patients with MASLD. **C.** Barplot analysis of the species level of the gut microbial community in the feces of healthy individuals and patients with MASLD. The dots show individual participant measurements. Participant numbers in each group are as follows: (Figure S7A-C) n=10.

Table S1. Characteristics of subjects with or without MASLD (no MASLD), related to Figure 7

MASLD			
Number	Gender	Age	BMI
MASLD1	man	30	36.8
MASLD2	woman	18	32
MASLD3	woman	16	47.7
MASLD4	woman	43	37.8
MASLD5	woman	23	37.1
MASLD6	man	42	37.3
MASLD7	woman	27	47.3
MASLD8	woman	38	38.2
MASLD9	woman	20	46.4
MASLD10	man	38	47
no MASLD			
Number	Gender	Age	BMI
Health1	man	38	35.2
Health2	woman	31	32.5
Health3	woman	28	31
Health4	woman	49	32.5
Health5	woman	49	24.4
Health6	woman	40	35.9
Health7	man	37	25.1
Health8	man	49	21.9
Health9	man	28	22.9
Health10	man	21	27.8

Table S2. Baseline characteristics of MASLD subjects in clinical therapy, related to Figure 7

Characteristic	S. cerevisiae (n=7)	Placebo (n=7)	Total (n=14)
Female/male (n)	1/6	3/4	4/10
Age (years)	50.0 ± 11.2	38.9 ± 8.1	44.4 ± 11.0
BMI (kg m-2)	26.67 ± 2.73	32.91 ± 14.22	29.79 ± 10.36
HbA1c (%)	9.47 ± 2.16	7.83 ± 2.07	8.65 ± 2.52
Fasting plasma glucose (mmol/L)	13.34 (8.20, 14.76)	6.25 (5.97, 8.66)	8.43 (6.14, 14.16)
Fasting serum insulin (μU/mL)	10.40 (2.45, 20.30)	6.22 (2.14, 9.45)	6.82 (2.45, 10.40)
HOMA-IR	4.32 (1.44, 12.04)	1.97 (0.82, 2.62)	2.30 (1.44, 4.32)
HOMA-β	41.26 (5.05, 44.26)	26.67 (8.29, 68.73)	33.96 (8.29, 60.08)
TG (mmol/L)	2.52 (1.18, 6.43)	1.87 (1.48, 4.13)	2.49 (1.48, 4.13)
TC (mmol/L)	6.30 ± 2.19	5.05 ± 1.39	5.68 ± 1.88
HDL-C (mmol/L)	1.24 (1.14, 1.65)	1.10 (0.89, 1.42)	1.24 (1.10, 1.52)
LDL-C (mmol/L)	3.56 ± 0.72	2.66 ± 0.63	3.11 ± 0.80
Severity of MASLD	1.0 (1.0, 3.0)	3.0 (1.8, 3.0)	3.0 (1.0, 3.0)

Table S3. Sequence of primers for Q-PCR, related to Methods.

Name	Sequence (5’ to 3’)
M-β-actin-For	CACTGTCGAGTCGCGTCC
M-β-actin-Rev	TCATCCATGGCGAACTGGTG
M-*Cpt1a*-For	TGGCATCATCACTGGTGTGTT
M-*Cpt1a*-Rev	GTCTAGGGTCCGATTGATCTTTG
M-*Mcad*-For	AACACAACACTCGAAAGCGG
M-*Mcad*-Rev	TTCTGCTGTTCCGTCAACTCA
M-*Acat1*-For	CAGGAAGTAAGATGCCTGGAAC
M-*Acat1*-Rev	TGCAGCAGTACCAAGTTTAGTG
M-*Acox1*-For	TAACTTCCTCACTCGAAGCCA
M-*Acox1*-Rev	AGTTCCATGACCCATCTCTGTC
M-*Fads2*-For	GGCCACTTAAAGGGTGCCTC
M-*Fads2*-Rev	GGCTCTTTATGTCCGGGTCC
M-*Scd1*-For	GCTGAACACCCATCCCGAG
M-*Scd1*-Rev	TGTAAGAACTGGAGATCTCTTGGA
M-*Mttp*-For	TCAGATGGACGCCAGCTTTT
M-*Mttp*-Rev	GCCAGTTGTGTGACCGCTAT
M-*Apob*-For	TACTTCCACCCACAGTCCCCT
M-*Apob*-Rev	CCTTAGAAGCCTTGGGCACAT
M-*CD36*-For	TGTGGAGCAACTGGTGGATG
M-*CD36*-Rev	CGTGGCCCGGTTCTACTAAT
M-*Fatp2*-For	CAATGCCCCTGTACCACAGT
M-*Fatp2*-Rev	CGGTCATTTGGTTTCTGCGG
M-*Ahr*-For	GCCCTTCCCGCAAGATGTTAT
M-*Ahr*-Rev	TCAGCAGGGGTGGACTTTAAT
M-*Cyp1a1*-For	CAATGAGTTTGGGGAGGTTACTG
M-*Cyp1a1*-Rev	CCCTTCTCAAATGTCCTGTAGTG
H-*Tph1*-For	CTACTGGCGCCCGAGTTTTA
H-*Tph1*-Rev	ATGAGACTTGCTCTTCCCCT
H-*Aaad*-For	CTCCCGGCTGCCTTTTTCA
H-*Aaad*-Rev	CTCCTTCCCTCTCCTTCGGA
H-*Mao-a*-For	CCACTCCTGTGCCTACGAC
H-*Mao-a*-Rev	TGGCAGCAGATAGTCCTGAA
H-*Mao-a*-For (for ChIP-qPCR)	GAGCTGAGGGAGTTGAGGTG
H-*Mao-a*-For (for ChIP-qPCR)	CTGCTCTCCACCCTGATCTC
18S rDNA-For	ATTGGAGGGCAAGTCTGGTG
18S rDNA-Rev	CCGATCCCTAGTCGGCATAG
16S rDNA-For	AGAGTTTGATCMTGGCTCAG
16S rDNA-Rev	CTGCTGCCTYCCGTA
Saccharomyces cerevisiae-For	TTAAGTGCGCGGTCTTGCTA
Saccharomyces cerevisiae-Rev	GTATCGCATTTCGCTGCGTT

REAGENT or RESOURCE	SOURCE	IDENTIFIER	PURCHASE DATE
Antibodies			
CPT1A Polyclonal antibody	Proteintech	Cat# 15184-1-AP RRID:AB_2084676	July 2024
ACAT1 Polyclonal antibody	Proteintech	Cat# 16215-1-AP RRID:AB_2220210	July 2024
ACOX1 Monoclonal antibody	Proteintech	Cat# 68017-1-Ig AB_2918763	July 2024
GAPDH Monoclonal antibody	Proteintech	Cat# 60004-1-Ig RRID:AB_2107436	July 2024
Anti-ACADM/MCAD antibody	Abcam	Cat# ab92461 RRID:AB_10563530	July 2024
Phospho-NF-κB p65 (Ser468) Recombinant monoclonal antibody	Proteintech	Cat# 82335-1-RR RRID:AB_3083091	February 2025
NF-κB p65 Recombinant monoclonal antibody	Proteintech	Cat# 80979-1-RR RRID:AB_2918923	February 2025
Phospho-IkB Alpha (Ser32/36) Recombinant monoclonal antibody	Proteintech	Cat# 82349-1-RR RRID:AB_3073626	February 2025
IkB Alpha Polyclonal antibody	Proteintech	Cat# 10268-1-AP RRID:AB_2151423	February 2025
AHR Monoclonal antibody	Proteintech	Cat# 67785-1-Ig RRID:AB_291854	Aprial 2025
CoraLite488-conjugated Goat Anti-Mouse IgG(H+L)	Proteintech	Cat# SA00013-1 RRID:AB_2810983	Aprial 2025
Histone H3 Polyclonal antibody	Proteintech	Cat# 17168-1-AP RRID:AB_2716755	Aprial 2025
Alpha Tubulin Polyclonal antibody	Proteintech	Cat# 11224-1-AP RRID:AB_2210206	Aprial 2025
Experimental models: Cell lines			
AML-12	ATCC	CRL-2254; RRID:CVCL_0140	August 2024
Caco-2	ATCC	HTB-37; RRID:CVCL_0025	March 2023
HEK-293T	ATCC	CRL-3216; RRID:CVCL_0063	June 2020
Experimental models: Organisms/strains			
Mouse: C57BL/6J	Institute of Model Animals, Nanjing University	RRID:IMSR_JAX:000664	N/A
Mouse: Card9$^{-/-}$	Dr. Xin Lin (Tsinghua University	N/A	August 2024
Mouse:Tlr1$^{-/-}$	GemPharmatech Co., Ltd	C57BL/6JGpt-Tlr1em3Cd3810in1/Gpt,Strain NO. T006732	August 2024
Chemicals, peptides, and recombinant proteins			
DMEM	Gibco	Cat# 11965092	August 2024
DMEM/F-12	Gibco	Cat# 11320033	August 2024
Opti-MEM medium	Gibco	Cat# 31985070	January 2025
QIAamp® Fast DNA Stool Mini Kit	Qiagen	Cat# 51604	November 2024
DAPI Fluoromount-G	SouthernBiotech	Cat# 0100-20	February 2025
L-Tryptophan	Mce	Cat# HY-N0623	February 2025

Free Fatty Acids	Sigma	Cat# P5585-10G	March 2025
Clorgyline	Mce	Cat# HY-14197	May 2025
Lipofectamine 2000	Invitrogen	Cat# 11668019-1.5mL	January 2025
BODIPY™ 493/503	Invitrogen	Cat# D3922	February 2025
FastPure Cell/Tissue Total RNA Isolation Kit V2	Vazyme	Cat# RC112-01	June 2024
HiScript III RT SuperMix for qPCR	Vazyme	Cat# R323-01	June 2024
180 kDa Prestained Protein Marker	Vazyme	Cat# MP102-01	May 2024
PowerUp™ SYBR™ Green	Applied Biosystems	Cat# A25742	June 2024
RIPA Buffer (High Strength)	Fudebio-tech	Cat# FD009	June 2024
Phosphatase Inhibitor Cocktail III	Mce	Cat# HY-K0023	March 2024
Triton X-100	Sigma-Aldrich	Cat# SLBT3016	May 2024
4% Paraformaldehyde Fixative	Biosharp	Cat# BL539A	June 2024
Beyotime Enhanced BCA Protein Assay Kit	Beyotime	Cat# P0010	May 2024
Vancomycin	Mce	Cat# HY-B0671	January 2025
Ampicillin	Mce	Cat# HY-B0522	January 2025
Neomycin sulfate	Mce	Cat# HY-B0470	January 2025
Metronidazole	Mce	Cat# HY-B0318	January 2025
Amphotericin B	Mce	Cat# HY-B0221	January 2025
Critical commercial assays			
Seahorse XF Cell Mito Stress Test Kit	Agilent Technologies	Cat# 103010-100	January 2025
Aspartate aminotransferase Assay Kit	Nanjing Jiancheng Institute of Biotechnolgy	Cat# C010-2-1	July 2024
Alanine aminotransferase Assay Kit	Nanjing Jiancheng Institute of Biotechnolgy	Cat# C009-2-1	July 2024
Triglyceride assay kit	Nanjing Jiancheng Institute of Biotechnolgy	Cat# A110-1-1	July 2024
Total cholesterol assay kit	Nanjing Jiancheng Institute of Biotechnolgy	Cat# A111-1-1	July 2024
Nonesterified Free fatty acids assay kit	Nanjing Jiancheng Institute of Biotechnolgy	Cat# A042-2-1	January 2025
Free Fatty Acids(FFA)Content Assay Kit	Solarbio	Cat# BC0595	January 2025
5-HIAA ELISA Kit	Elabscience	Cat# E-EL-0075c	February 2025
ST/5-HT ELISA Kit	Elabscience	Cat# E-EL-0033c	February 2025
Luciferase activity	Promega	Cat# E1910	March 2025
Cell Counting Kit-8	Dojindo	Cat# CK04	January 2025

135

Applied Biosystems
GeneMapper ID v3.2

Sample File	Sample Name	Panel	OS	SQ
11_C02_CellLineAuthentication-1--0630.fsa	293T	21Plex_STR_Panel_v1.1		

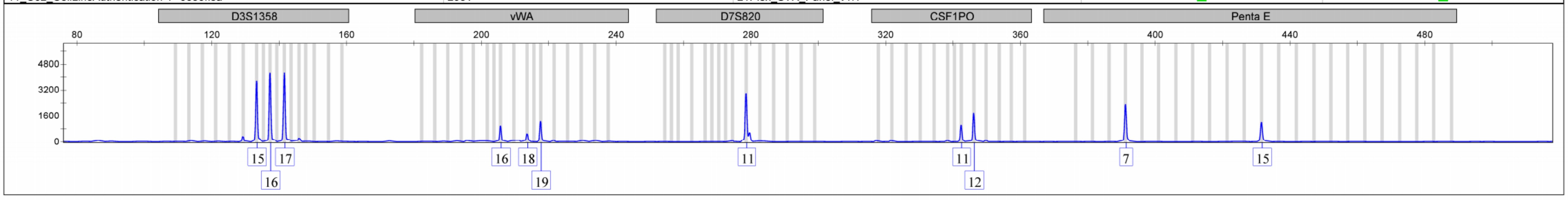

11_C02_CellLineAuthentication-1--0630.fsa | 293T | 21Plex_STR_Panel_v1.1

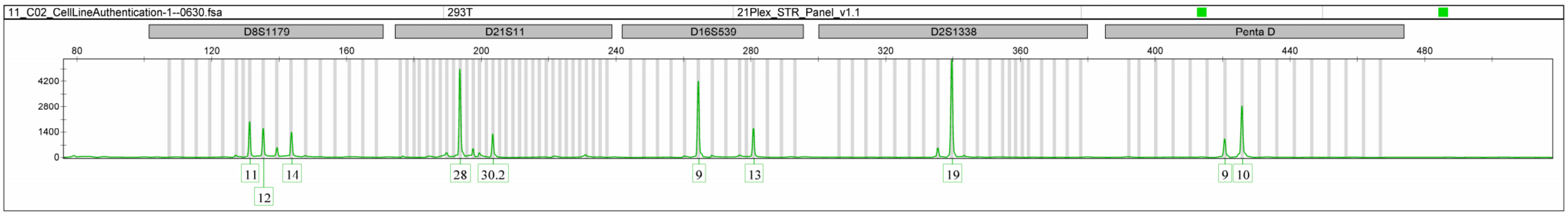

11_C02_CellLineAuthentication-1--0630.fsa | 293T | 21Plex_STR_Panel_v1.1

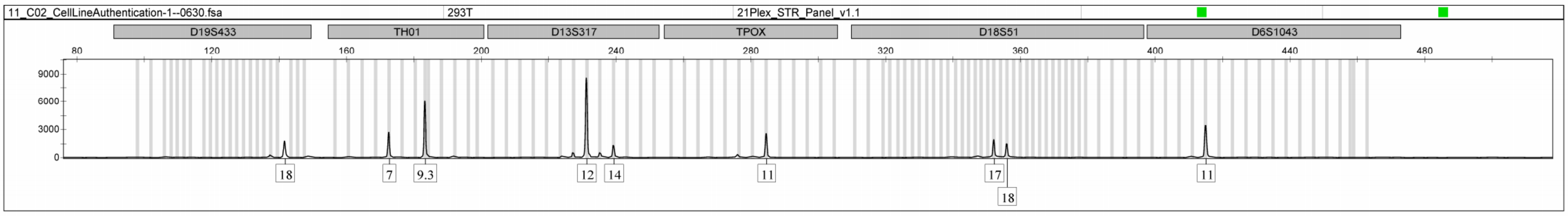

11_C02_CellLineAuthentication-1--0630.fsa | 293T | 21Plex_STR_Panel_v1.1

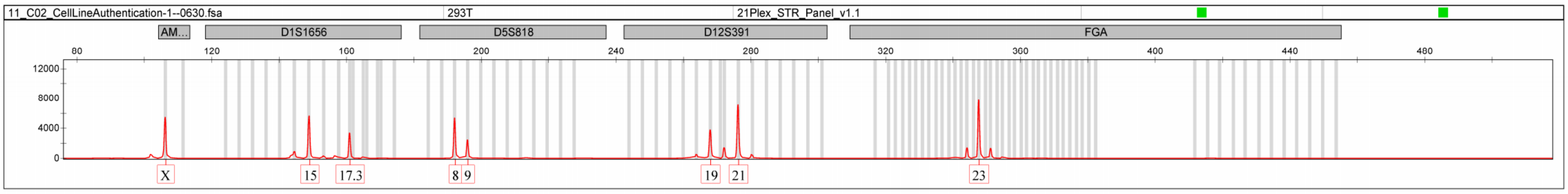

Sample File	Sample Name	Panel	SQO	OS	SQ
86_F11_Cellidentification-1-0331.fsa	CACO2	21Plex_STR_Panel_v1.2			

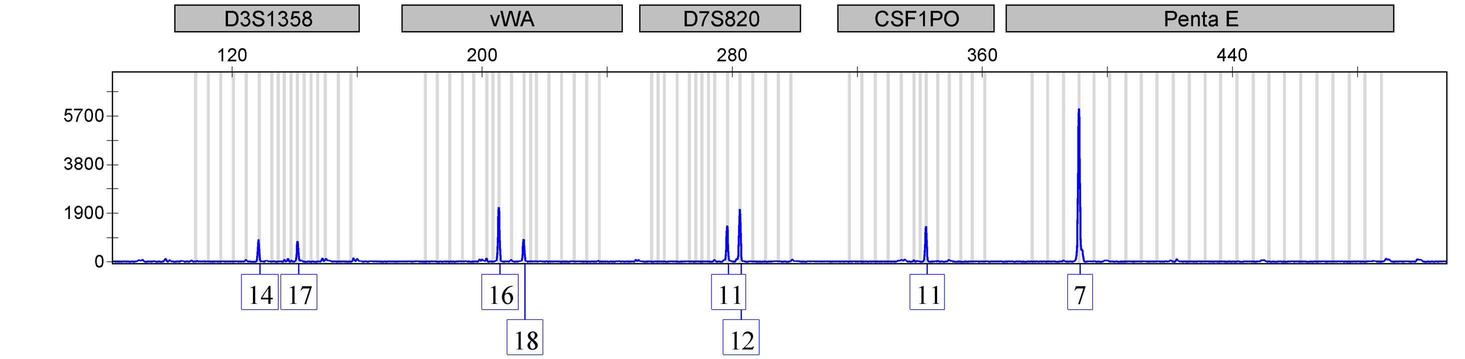

Sample File	Sample Name	Panel	SQO	OS	SQ
86_F11_Cellidentification-1-0331.fsa	CACO2	21Plex_STR_Panel_v1.2			

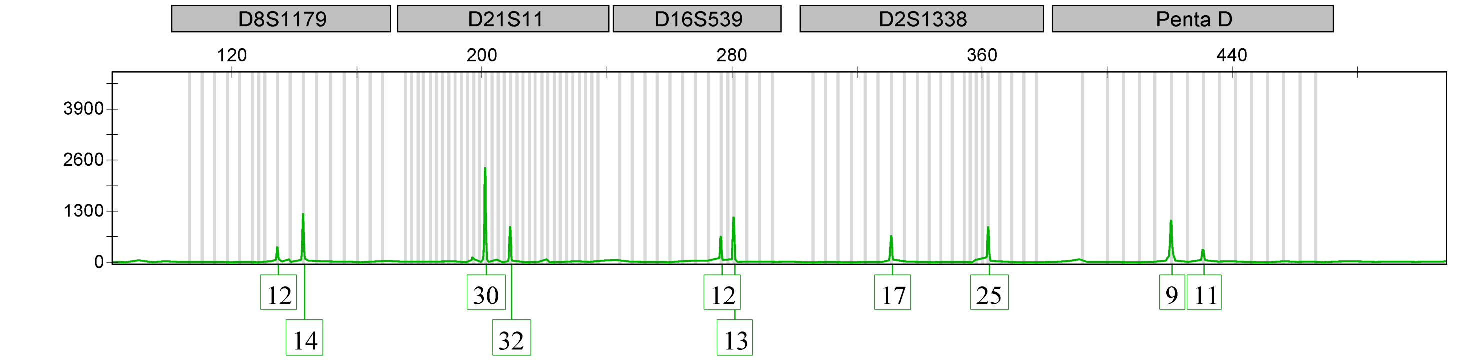

Sample File	Sample Name	Panel	SQO	OS	SQ
86_F11_Cellidentification-1-0331.fsa	CACO2	21Plex_STR_Panel_v1.2			

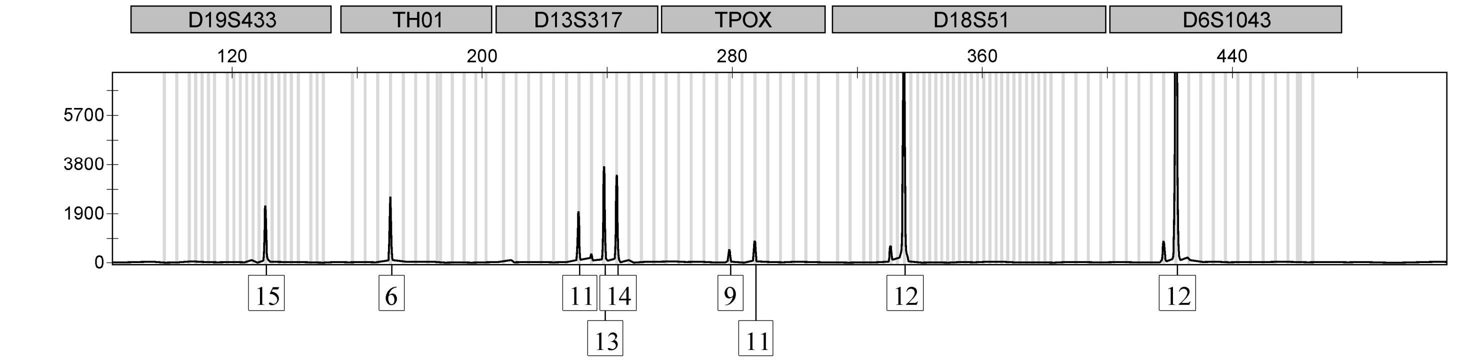

Sample File	Sample Name	Panel	SQO	OS	SQ
86_F11_Cellidentification-1-0331.fsa	CACO2	21Plex_STR_Panel_v1.2			

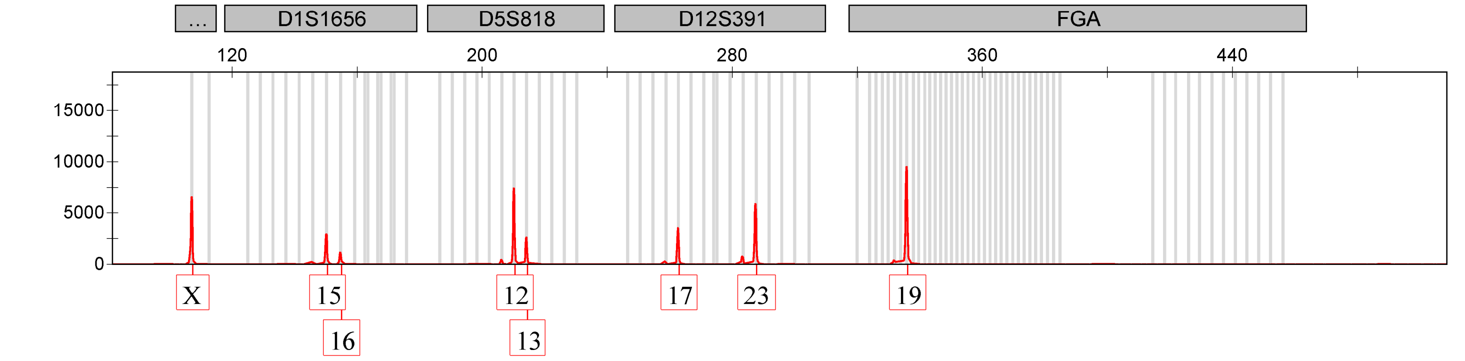

Applied Biosystems

GeneMapper ID v3.2

Sample File	Sample Name	Panel	SQO	OS	SQ
45_E06_Cellidentification-2----0815.fsa	AML12	shu-1-dup		■	▲

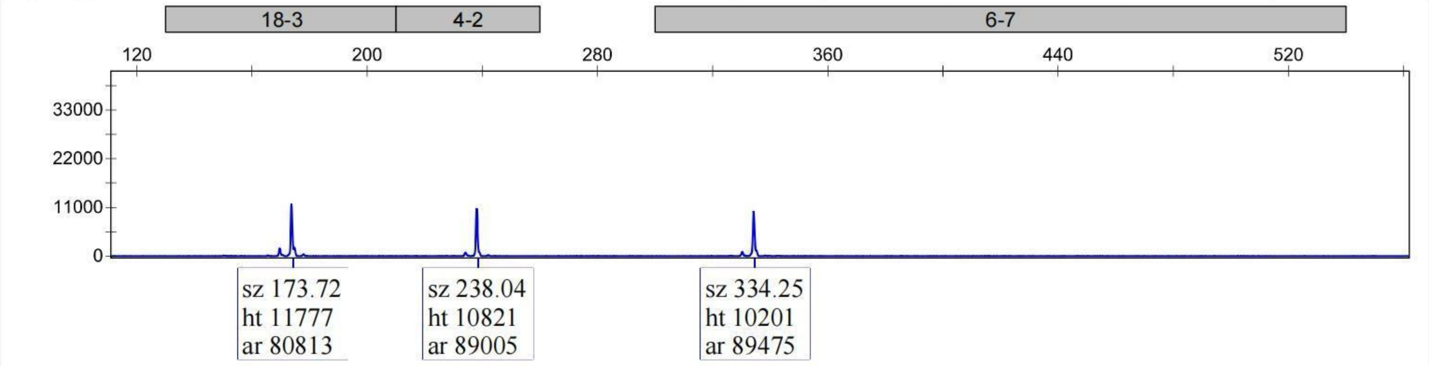

Sample File	Sample Name	Panel	SQO	OS	SQ
45_E06_Cellidentification-2----0815.fsa	AML12	shu-1-dup		■	▲

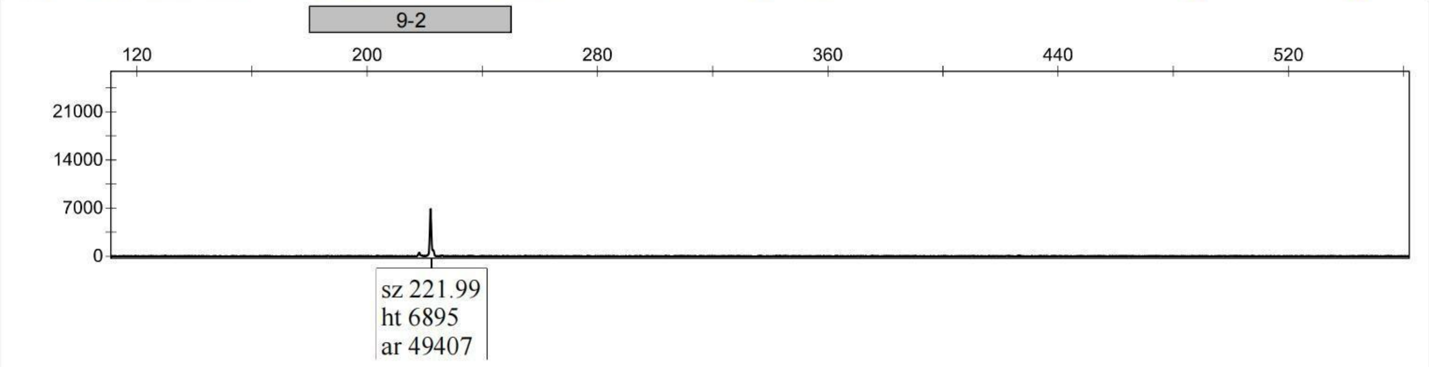

Sample File	Sample Name	Panel	SQO	OS	SQ
61_E08_Cellidentification-2----0815.fsa	AML12	shu-2-dup		■	▲

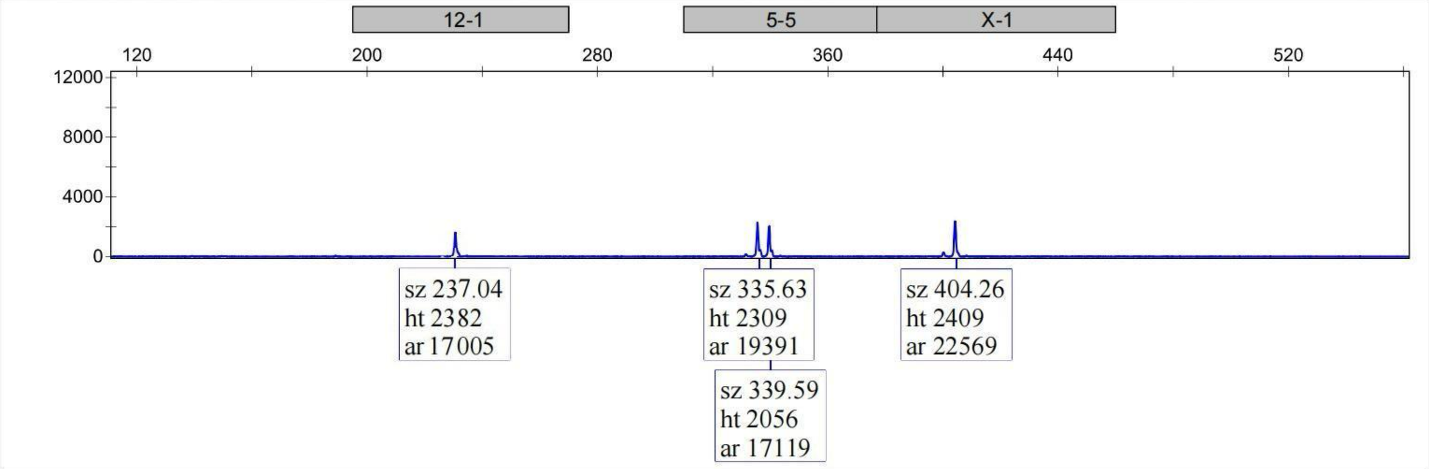

Applied Biosystems
GeneMapper ID v3.2

Sample File	Sample Name	Panel	SQO	OS	SQ
61_E08_Cellidentification-2----0815.fsa	AML12	shu-2-dup			

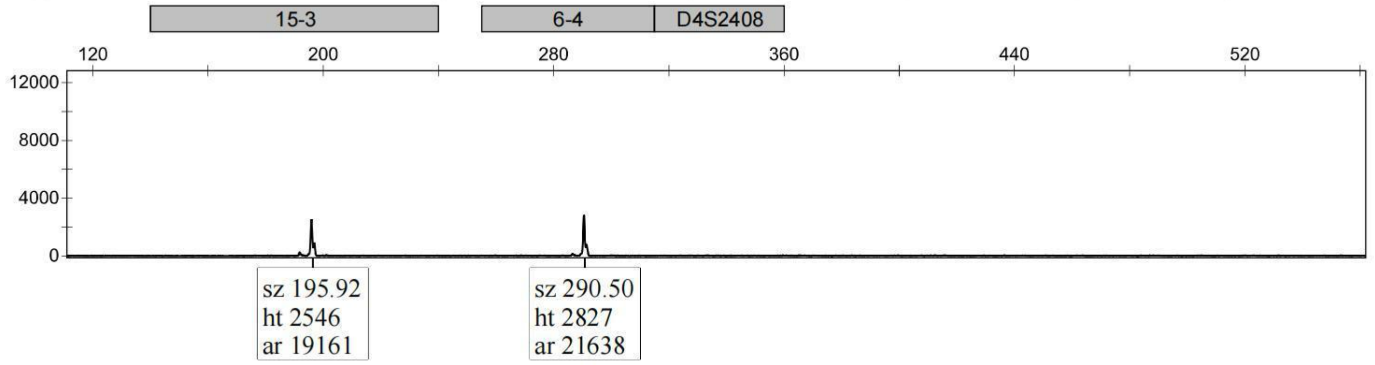

Supplement: Supplementary file 1 — Supporting File 1: advs75268‐sup‐0001‐SuppMat.pdf. [file ADVS-13-e14830-s002.pdf]
